# Elucidation of the di-c-glycosylation steps during biosynthesis of the antitumor antibiotic, kidamycin

**DOI:** 10.3389/fbioe.2022.985696

**Published:** 2022-08-25

**Authors:** Kyung Taek Heo, Byeongsan Lee, Jae-Hyuk Jang, Young-Soo Hong

**Affiliations:** ^1^ Chemical Biology Research Center, Korea Research Institute of Bioscience and Biotechnology, Chungbuk, South Korea; ^2^ Department of Bio-Molecular Science, KRIBB School of Bioscience, University of Science and Technology(UST), Daejeon, South Korea

**Keywords:** kidamycin, biosynthetic gene cluster (BGC), C-glycosyltransferase, methyltransferase, angucycline, streptomyces

## Abstract

Kidamycins belong to the pluramycin family of antitumor antibiotics that contain di-C-glycosylated angucycline. Owing to its interesting biological activity, several synthetic derivatives of kidamycins are currently being developed. However, the synthesis of these complex structural compounds with unusual C-glycosylated residues is difficult. In the kidamycin-producing *Streptomyces* sp. W2061 strain, the genes encoding the biosynthetic enzymes responsible for the structural features of kidamycin were identified. Two glycosyltransferase-coding genes, *kid7* and *kid21*, were found in the kidamycin biosynthetic gene cluster (BGC). Gene inactivation studies revealed that the subsequent glycosylation steps occurred in a sequential manner, in which Kid7 first attached N,N-dimethylvancosamine to the C10 position of angucycline aglycone, following which Kid21 transferred an anglosamine moiety to C8 of the C10-glycosylated angucycline. Therefore, this is the first report to reveal the sequential biosynthetic steps of the unique C-glycosylated amino-deoxyhexoses of kidamycin. Additionally, we confirmed that all three methyltransferases (Kid4, Kid9, and Kid24) present in this BGC were involved in the biosynthesis of these amino-deoxyhexoses, N,N-dimethylvancosamine and anglosamine. Aglycone compounds and the mono-C-glycosylated compound obtained in this process will be used as substrates for the development of synthetic derivatives in the future.

## 1 Introduction

**FIGURE 1:**
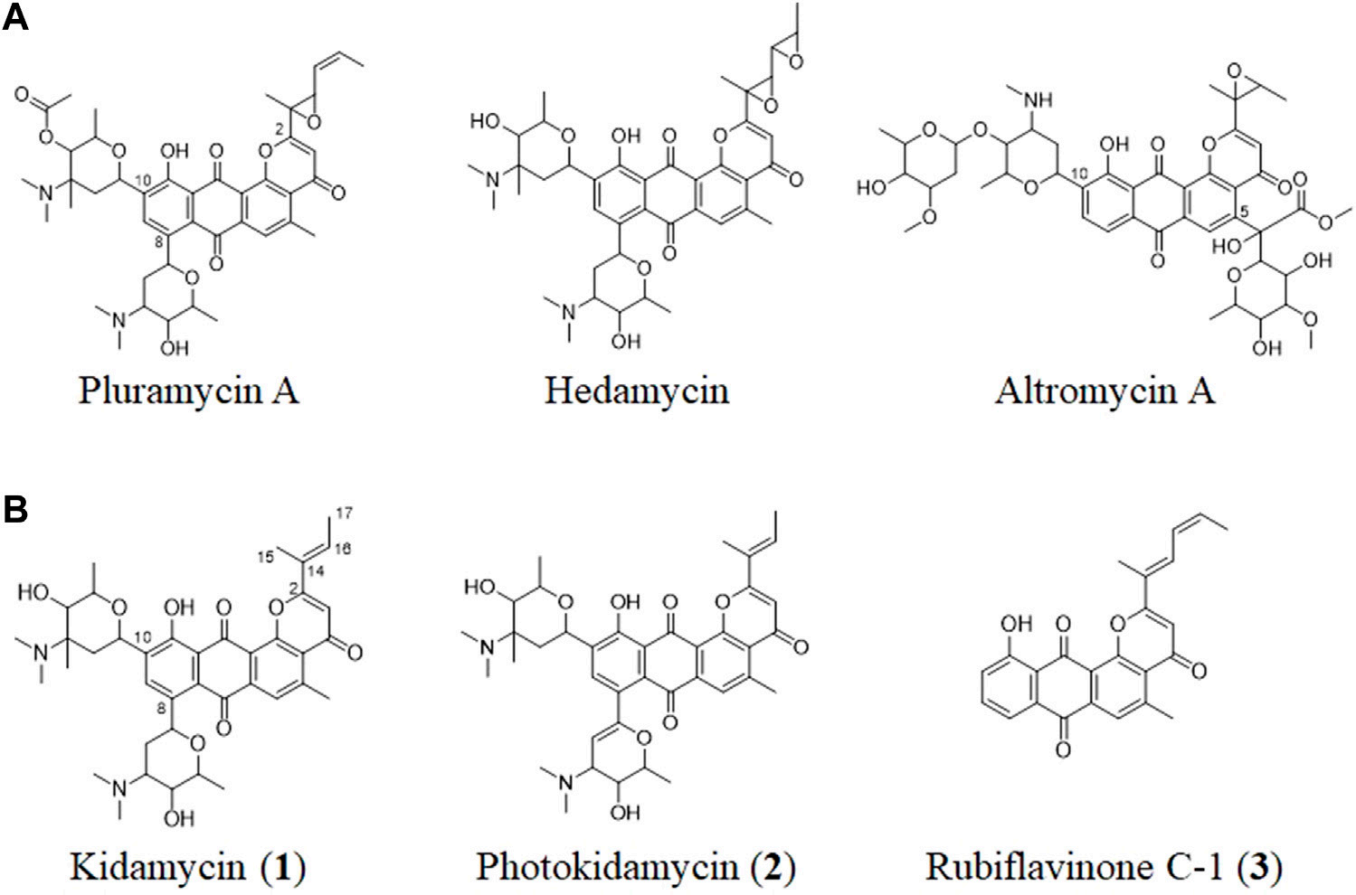
**(A)** Structures of representative pluramycin family angucyclines and **(B)** the kidamycin (**1**), photokidamycin (**2**), and rubiflavinone C-1 (**3**) isolated from *Streptomyces* sp. W2061.

Angucycline compounds of the pluramycin family are a group of naturally occurring antibiotics with antitumor activity (Hansen and Hurley, 1996; Kharel et al., 2012). They contain a 4*H*-anthra (1,2-*b*)pyran-4,7,12-trione substructure with branched side chain at C2, as well as C-glycoside moieties (Figure 1). These compounds intercalate with the minor groove of DNA via sugar-mediated interactions and direct alkylation of guanine in the major groove in a sequence-specific manner (Hansen et al., 1995).

The pluramycin family compounds are divided into two subfamilies that represent different C-glycosylated patterns at C10 and C5 or C8 positions. The classical pluramycins are represented by hedamycin, which contains two amino-deoxyhexoses, an anglosamine sugar attached to the C8 position and a N,N-dimethylvancosamine group attached to the C10 position; and the altromycins, containing altroses at the C5 position (Figure 1). Rubifavinone and sapurimycin are the simplest analogs of the pluramycin family, which lack sugar substitution (Hara et al., 1991; Schumacher et al., 1995; Harunari et al., 2022). Several studies have shown that the selectivity of the pluramycin family compounds correlate with the sugar substitution pattern at the C5, C8, and C10 positions (Prakash et al., 1995; Hansen and Hurley, 1996). Based on this observation, several stereo- and regio-selective glycosylated synthetic derivatives have been developed recently (Kitamura et al., 2014a; Kitamura et al., 2014b; Hartung et al., 2014).

Kidamycin is one of the earliest known members of the pluramycin family (Kanda, 1971; Furukawa et al., 1975). Kidamycin also exhibits cytotoxic activity against various tumors (Rixson et al., 2015). The photokidamycin as well as kidamycin show selective activity on MDA-MB-231, a triple-negative breast cancer cell line (Cho et al., 2019). Although kidamycin consists of planar polycyclic angucycline identical to that of hedamycin, the structure of the subgroups differs because of the presence of a 2-butenyl residue at C2. It also harbors two aminosugars, angolosamine and N,N-dimethylvancosamine, branched at C8 and C10, respectively, attached *via* C-glycosidic bonds. However, the genetic and biochemical basis of kidamycin biosynthesis is still unknown and details of the *di-*C-glycosylation biosynthetic step has not been proposed yet.

Herein we report the cloning and characterization of the biosynthetic gene cluster (BGC) responsible for the biosynthesis of kidamycin and its aglycone with different residues at C2, which are produced by *Streptomyces* sp. W2061. The BGC for kidamycin (*kid*) includes enzymes that are responsible for the biosynthesis of the core polyketide backbone (Kid12–20), nucleotidyl-activated aminosugar moieties (Kid4-9 and Kid21-28), and two glycosyltransferases (GTs; Kid7 and Kid21). Gene inactivation studies revealed that the subsequent glycosylation steps occurred in a sequential manner: Kid7 first attached the N,N-dimethylvancosamine moiety to C10 of the angucycline aglycone; and Kid21 then transferred an angolosamine moiety to C8 of the *mono* C-glycosylated angucycline core. In addition, we confirmed that all three methyltransferases (Kid4, Kid9, and Kid24) were involved in the biosynthesis of the aminosugar moieties.

## 2 Materials and methods

### 2.1 Plasmids, strains, culture conditions, and extraction

Antibiotics were added to the medium at the following concentrations: apramycin, 50 mg/L; kanamycin, 50 mg/L; and chloramphenicol, 25 mg/L. The restriction enzymes (NEB, United States; Takara, Japan), KOD-plus-DNA polymerase (Toyobo, Japan), PrimeSTAR^®^ GXL DNA polymerase (Takara, Japan) and DNA ligation kit (Takara, Japan) were used according to the manufacturers’ manuals. T-Blunt vector (BioFact, Deajeon, Korea) was used to clone the polymerase chain reaction (PCR) products. Gene inactivation experiments were performed using the vector pKC1139 (Kieser et al., 2000), and the kanamycin resistance gene from pFD-NEO-S (Denis and Brzezinski, 1991) was used as the selection marker. *Escherichia coli* DH5α was used for plasmid cloning and amplification, and ET12567/pUZ8002 was used for introducing the plasmid into *Streptomyces* sp. W2061 strain via conjugation. All bacterial strains and plasmids used in this work are summarized in Supplementary Table S1.

*Streptomyces* sp. W2061 and mutant strains were grown in ISP4 plate (10 g/L soluble starch, 1 g/L K_2_HPO_4_, 1 g/L MgSO_4_·7H_2_O, 1 g/L NaCl, 2 g/L (NH_4_)_2_SO_4_, 2 g/L CaCO_3_, 0.001 g/L FeSO_4_·7H_2_O, 0.001 g/L MnCl_2_·4H_2_O, 0.001 g/L ZnSO_4_·7H_2_O, and 15 g/L agar, pH 7.0–7.4) at 28°C for 4 days. Then, they were inoculated into seed culture M2 medium (2 g/L yeast extract, 5 g/L glucose, 25 ml/L glycerol, 4 g/L soytone, 0.03 g/L CaCO_3_, and pH 7.2) and incubated for 2 days at 28°C, following which 15 ml seed culture was transferred to 1 L flask containing 300 ml of M2X medium (M2 medium + 5 g/L MgCO_3_) and incubated for 5–7 days. For compounds isolation for NMR-accessible amounts from the present culture conditions, 30 flasks containing 300 ml of M2X medium were used. The culture broth was extracted with equal volume of ethyl acetate; next, ethyl acetate was dried and the extract was resuspended in methanol for high performance liquid chromatography (HPLC) and liquid chromatography-mass spectrometry (LC-MS) analysis.

For bioconversion, the ΔKid7 mutant was grown in ISP4 plate at 28°C for 4 days and then inoculated into seed culture M2 medium. After 2 days at 28°C, 15 ml of the seed culture was added to a 1-L flask containing 300 ml M2X medium and incubated at 28°C. Compound **7** was added after 3 days and incubated for 4 days. The extract was prepared as mentioned above and analyzed using LC-MS.

### 2.2 Bioinformatics analysis

The genomic DNA of *Streptomyces* W2061 was obtained and sequenced by PacBio RSⅡ sequencer (Pacific BioSciences, Menlo Park, CA, United States). The sequence raw data were assembled using SMRT Analysis (v2.3.0 HGAP.2) and predicted using the BGC New Genome Annotation System (Newgas, Genotech, Korea) and antiSMASH (Blin et al., 2021). The amino acid sequences of Kid7, Kid21, and other GTs involved in the generation of natural products by *Streptomyces* [AknK (AAF70102), AknS (AAF73455), AraGT (ABL09968), Asm25 (AAM54103), DesVII (Q9ZGH7), ElaGT (ADP68587), EryBV (AAB84072), EryCIII (A4F7P3), GilGT (AAP69578), Gra-ORF14 (CAA09635), HedJ (AA85354), HedL (AAP85354), LanGT (AAD13562), LanGT2 (AAD13553), Med8 (BAC79040), SsfS6 (ADE34512), SunS (KIX81208), UrdGT2 (AAF00209) and VlnC (BAJ52701)] were aligned using the Clustal W method. The amino acid sequences of the kidamycin genes are described in the Supporting Information. Phylogenetic tree analysis using the minimum evolution method was based on the results of sequence alignment, and evolutionary distances were computed using the Poisson correction method in the MEGA7 software (Kumar et al., 2016).

### 2.3 Construction of disruption vectors and mutant strains

The gene knockout recombinants were generated using homologous recombination. The target region was replaced with a kanamycin resistance gene. The kanamycin resistance cassette was digested with KpnI and PstI or SalI from plasmid pFD-NEO-S (Denis and Brzezinski, 1991). Two homologous regions were amplified using the appropriate primers (Supplementary Table S1). For constructing all gene replacement vectors, three fragments were ligated with plasmid pKC1139 and introduced into *Streptomyces* sp. W2061 strain via conjugation from *E. coli* ET12567/pUZ8002. The exoconjugants were selected based on antibiotic resistance (kanamycin and apramycin) and PCR genotyping. The double-crossover mutants were screened based on kanamycin resistance and PCR genotyping (kid4_scF/R for ∆Kid4 mutant, kid7_scF/R for ∆Kid7 mutant, kid9_scF/R for ∆Kid9 mutant, kid21_scF/R for ∆Kid21 mutant, and kid24scF/R for ∆Kid24 mutant; Supplementary Table S2).

In particular, to inactivate *kid4,* a 1 kb EcoRI/KpnI fragment generated using kid4-H1-F and kid4-H1-R, and a 1.1 kb PstI/HindIII fragment generated using kid4-H2-F and Kid4-H2-R, were ligated and cloned into the EcoRI and HindIII sites of pKC1139 to yield pKC-Kid4neo. To inactivate *kid7,* a 1.1 kb EcoR1/SalI fragment generated using kid7-H1-F and kid7-H1-R, and a 1.1 kb SalI/HindIII fragment generated using kid7-H2-F and Kid7-H2-R, were ligated and cloned in the EcoRI and HindIII sites of pKC1139 to yield pKC-Kid7neo. To inactivate *kid9,* a 1 kb EcoR1/KpnI fragment generated using kid9-H1-F and kid9-H1-R, and a 1.3 kb PstI/HindIII fragment generated using kid9-H2-F and Kid9-H2-R, were ligated and cloned in the EcoRI and HindIII sites of pKC1139 to yield pKC-Kid9neo. To inactivate the *kid19*, a 1kb EcoR1/KpnI fragment generated using kid19-H1-F and kid19-H1-R and a 1.1kb PstI/HindIII fragment generated using kid19-H2-F and Kid19-H2-R were ligated and cloned in the EcoRI and HindIII sites of pKC1139 to yield pKC-Kid19neo. To inactivate the *kid21,* a 1.5 kb EcoR1/SalI fragment generated using kid21-H1-F and kid21-H1-R, and a 1.2 kb SalI/HindIII fragment generated using kid21-H2-F and Kid21-H2-R, were ligated and cloned in the EcoRI and HindIII sites of pKC1139 to yield pKC-Kid21neo. To inactivate *kid24*, a 1.4 kb EcoR1/SalI fragment generated using kid24-H1-F and kid24-H1-R, and a 1.3 kb SalI/HindIII fragment generated using kid24-H2-F and Kid24-H2-R, were ligated and cloned in the EcoRI and HindIII sites of pKC1139 to yield pKC-Kid24neo (Supplementary Table S1 and Supplementary Figure S1).

### 2.4 LC-MS analysis

The samples were dissolved in methanol and analyzed using a Thermo U3000-LTQ XL ion trap mass spectrometer (Thermo Scientific, Waltham, MA, United States) equipped with an electrospray ionization (ESI) mass source. Chromatographic separation of the compounds was achieved using a Waters HSS T3 C18 column (2.1 × 150 mm; 2.5 μm) at the flow rate of 0.3 ml/min. The mobile phases, A and B, contained 0.1% formic acid along with water and acetonitrile, respectively. Gradient elution was performed as follows: 5–100% B for 0–15 min with a linear gradient, followed by 5 min of 100% B. The MS/MS system was operated in ESI mode. The operating parameters were as follows: spray needle voltage, +5 kV; ion transfer capillary temperature, 275°C; nitrogen sheath gas, 35 (arbitrary units); auxiliary gas, 5 (arbitrary units). The ion trap contained helium damping gas, which was introduced in accordance with the manufacturer’s recommendations. Mass spectra were acquired in an *m/z* range of 100–2000, applying three microscans and a maximum ion injection time of 100 ms. Data-dependent mass spectrometry experiments were controlled using the menu-driven software provided in the Xcalibur system (version 4.0; Thermo Scientific).

### 2.5 Compound isolation

For isolation of compound **7**, the crude extract (7.3g) of *∆*Kid21 mutant was fractionated employing reversed-phase C18 vaccum column chromatography eluting with a stepwise MeOH:H_2_O solvent system of (20: 80 to 100: 0, each × 1 L). The 70 % (1043.4 mg) fraction was further fractionated using a CombiFlash RF (Teledyne ISCO, Lincoln, NE, United States) medium-pressure chromatography system (MPLC) on a Redisep RF C18 reverse-phase column under stepwise gradient elution with MeOH-H_2_O (from 20: 80, 40: 60, 60: 40, 80: 20 to 100: 0; 1 L for each step). MPLC fraction was separated using semipreparative HPLC (Waters Atlantis T3 C18 column: 10 × 250 mm, 5 μm) with an isocratic solvent system (45 % MeOH-H_2_O [0.05% trifluoroacetic acid (TFA)] over 20 min, UV 254 nm detection, flow rate: 3 ml/min) to obtain fraction containing compound **7** (26.0 mg). The separated fraction (26.0 mg) was further subjected to semipreparative HPLC (Waters Atlantis T3 C18 column (10 × 250 mm, 5 μm) a gradient solvent system (30% CH_3_CN-H_2_O (0.05 % TFA) to 100% CH_3_CN, 3 ml/min) over 20 min to yield compounds **7** (9.0 mg). The aglycone compounds **3**, **5**, and **6** are isolated by 2.1 mg, 4.2 mg, and 6.2 mg from *∆*Kid4 mutant, respectively. The other aglycone compound **4** (4.2 mg) is purified from *∆*Kid7 mutant. The structures of the purified compounds were determined based on ^1^H and ^13^C nuclear magnetic resonance (NMR) analysis, as well as HSQC, COSY and HMBC NMR experiments.

## 3 Results

### 3.1 Confirmation of the kidamycin BGC by disrupting the polyketide synthase gene

**FIGURE 2:**
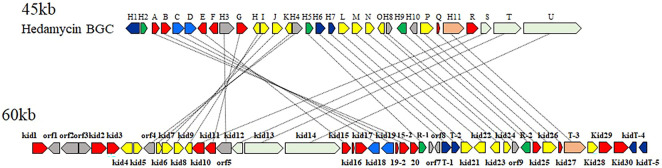
Comparison of the biosynthetic gene clusters encoding hedamycin (top) and kidamycin (bottom). Each arrow represents the direction of transcription of an open reading frame. Please see Supplementary Table S3 for complete cluster annotation.

Among the pluramycin family angucyclines, the mechanism of hedamycin biosynthesis, which involves the assembly of the angucycline core by a rare hybrid type I/type II PKS, has been studied extensively (Bililign et al., 2004; Das and Khosla, 2009). The structural similarities between kidamycin and hedamycin was indicative of substantial shared biosynthetic logic. We identified a putative kidamycin BGC (*kid*) in *Streptomyces* sp. W2061 using detailed bioinformatics analysis from draft whole genome sequencing data (Supplementary Table S3). The *kid* cluster is similar in content and organization to the known hedamycin BGC identified in *S. griseoruber* (Bililign et al., 2004) (Figure 2). The results of antiSMASH analysis revealed only one gene cluster including the hybrid type I/type II PKS, which was 87% similar to the hedamycin BGC. Sequence analysis of the 60 kb region revealed 59 open reading frames (ORFs), which included putative genes involved in the biosynthesis of the angucycline core, construction of angolosamine and N,N-dimethylvancosamine sugars, and tailoring of the core scaffold.

The structure of the kidamycin angucycline core is identical to that of hedamycin, except that its core has a 2-butenyl residue attached to C2 instead of the bisepoxide group found in hedamycin. We proposed that this kidamycin core group also arises from the use of a hybrid type I/type II PKS system. The *kid* cluster contains homologs of the type II PKS core components [chain length factor (CLF) Kid18, ketosynthase (KS) α Kid19, acyl carrier protein (ACP), Kid16] and tailoring enzymes [ketoreductase (KR) Kid20, cyclases Kid15, Kid15-1, and Kid19-1, and oxidase Kid27] required to construct an angucycline core polyketide (Supplementary Figure S2).

**FIGURE 3:**
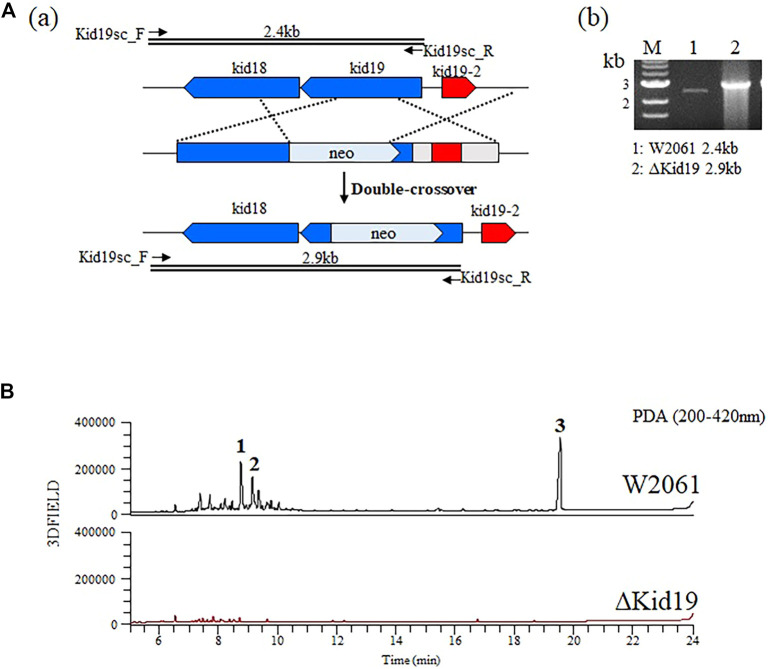
Strategies for gene disruption. **(A)** The ΔKid19 mutant resulted from a double crossover event to produce a kanamycin-resistant strain in which the *kid19* gene was disrupted (a). Confirmation of insertional *kid19* gene inactivation using PCR and the total genomic DNA of each mutant as the template (b). The primers (kid19sc_F & kid19sc_R; Supplementary Table S2) used to amplify the desired DNA fragments are indicated by solid arrows. M, 1 kb ladder; 1, wild type; 2, ΔKid19 mutant. **(B)** Comparative HPLC analysis of crude fermentation extracts showing production of kidamycins (**1** and **2**) and rubiflavinone C-1 (**3**) by strains W2061 and ΔKid19, revealing loss of kidamycins and rubiflavinone C-1 production due to the *kid19* gene deletion.

To validate that the *kid* gene cluster was indeed responsible for kidamycin biosynthesis, an insertional inactivation was generated with a kanamycin resistance marker to disrupt the function of the putative KSα, Kid19. The mutants were confirmed using PCR. A successful double crossover mutant, ∆Kid19, was confirmed using PCR and fermented parallel to the parent strain (Figure 3A). The ∆Kid19 mutant did not produce any detectable kidamycins or rubiflavinone C-1, confirming the role of Kid19 and the *kid* gene cluster in the production of these products (Figure 3B). Therefore, this *kid* BGC, at least the type II PKSs, play an important role in angucycline core biosynthesis, indicating that they are used together in the production of angucycline compounds with two different side chains, kidamycin and rubiflavinone C-1.

### 3.2 Sequence analysis of two putative C-glycosyl transferases (GTs) in the *kid* BGC

Kidamycin is characterized by the C-glycosidic attachment of two amino sugars to the angucycline core, a process anticipated to require GTs. After formation of angucycline core polyketides, a complex series of glycosylation events is required for generation of the final di-C-glycosidic structure. We identified 12 putative genes (*kid4*–*kid9*, *kid21*-*24*, *26,* and *28*) in the *kid* BGC, consistent with the biosynthesis of two amino sugar moieties and subsequent attachment to the angucycline core (Figure 2 and Supplementary Table S3).

**FIGURE 4:**
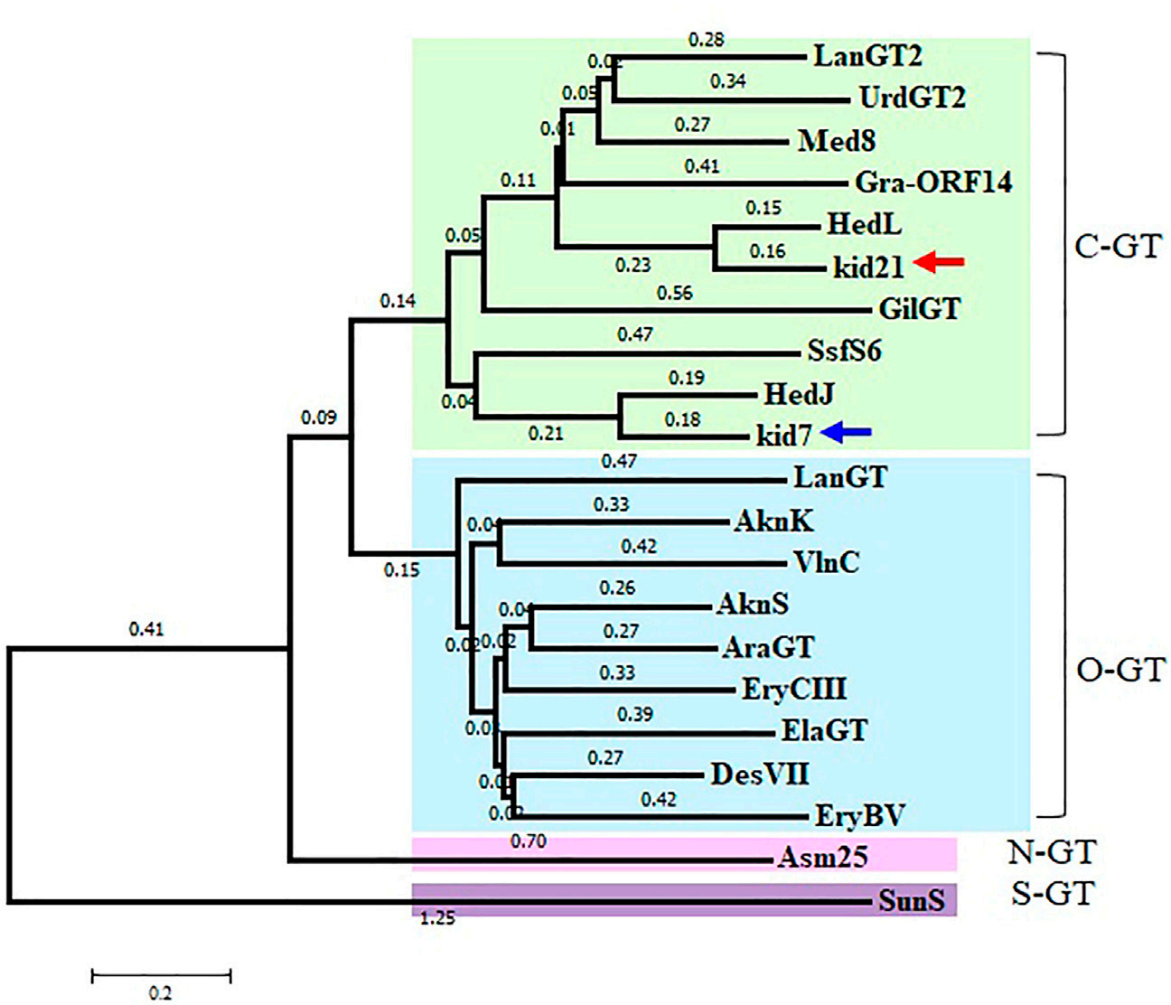
Phylogenetic analysis of 2 GTs, Kid7 and Kid21, compared to known GTs from *Streptomyces.* Arrow indicates the 2 GTs of this study. The sources and GenBank accession numbers of GTs involved in natural product biosynthesis by *Streptomyces*: AknK (AAF70102), AknS (AAF73455), AraGT (ABL09968), Asm25 (AAM54103), DesVII (Q9ZGH7), ElaGT (ADP68587), EryBV (AAB84072), EryCIII (A4F7P3), GilGT (AAP69578), Gra-ORF14 (CAA09635), HedJ (AA85354), HedL (AAP85354), LanGT (AAD13562), LanGT2 (AAD13553), Med8 (BAC79040), SsfS6 (ADE34512), SunS (KIX81208), UrdGT2 (AAF00209), and VlnC (BAJ52701). Amino acid sequences were aligned using the ClustalW method and the phylogenetic tree analysis was performed based on the sequence alignment using the Minimum Evolution method in the MEGA7 software.

Identification of 2 GTs in the *kid* BGC met the general requirement of one GT-one sugar for the sugar moieties of microbial natural products. Phylogenetic tree analysis of Kid7 and Kid21 indicated their involvement in C-glycosylation (Figure 4). Kid7 and Kid21, which are located far away from the polyketide core genes, shows significant homology to the C-GT found in the hedamycin BGC (HedJ and HedL) (Figure 4). Alignment of the amino acid sequences of Kid7 and Kid21 with those of HedJ and HedL, respectively, also showed that the proteins are significantly identical (Kid7 vs. HedJ, 67.5% similarity; Kid21 vs. HedL, 71.8% similarity) (Supplementary Table S4). Additionally, both Kid7 and Kid21 resemble Med-8 (52% and 59% similarity, respectively), a C-GT responsible for angolosamine transfer in medermycin biosynthesis (Ichinose et al., 2003; Cai et al., 2021). However, Kid21 is believed to be responsible for angolosamine transfer, because of its high degree of identity with Med-8.

### 3.3 Identification of sequential C-glycosylation steps in kidamycin biosynthesis

The function of Kid21 was elucidated using targeted gene inactivation. The pKC-kid21neo plasmid carrying the kanamycin resistance cassette within *kid21* was used to replace the chromosomal allele of this gene in *Streptomyces* sp. W2061 via a double crossover event. The mutation was confirmed using PCR (Supplementary Figure S1).

**FIGURE 5:**
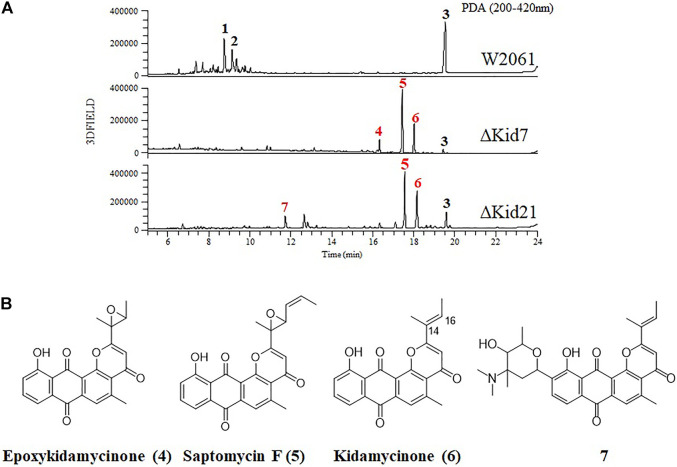
Disruption of *kid7* and *kid 21* gene loci. **(A)** HPLC analysis of the products of *Streptomyces* sp. W2061 (upper), ∆Kid7 (middle), and ∆Kid21 (bottom). **(B)** Structure of compounds epoxykidamycinone (**4**), saptomycin F (**5**), kidamycinone (**6**), and **7** isolated from the mutants.

Kidamycins were not detected in the extracts of the ∆Kid21 mutant. Instead, several new peaks were detected in the extract from the mutant strain, which were absent in the extract of the wild-type strain. However, the aglycone compound, rubiflavinone C-1 (**3**), identified in the parent strain, was still produced by the ∆Kid21 mutant (Figure 5). The ESI-MS spectrum of the new peak at 11.5 min (**7**) was generated at the *m/z* 532 [M + H]^+^ (Supplementary Figure S3), which was the same as that of the N,N-dimethylvancosamine moiety attached to the aglycone of kidamycin (kidamycinone). In particular, ions with *m/z* = 361 [M + H]^+^, indicating loss of the N,N-dimethylvancosamine moiety (171 Da), appeared at the MS/MS fragmentation profile of the ion with *m/z* = 532. This indicated that the product was N,N-dimethylvancosamine glycosylated kidamycinone. The new peaks that appeared in the ∆Kid21 mutant were purified from a 9 L fermentation and the chemical structure was obtained using NMR. The structure of **7** was confirmed to be that of N,N-dimethylvancosamine C10 glycosylated kidamycinone based on a comparison of the 1D and 2D NMR spectroscopic data of kidamycin with previously reported data (Supplementary Table S5 and Supplementary Figure S4–12). The NMR spectroscopic data of **7** were similar to those of kidamycin, except that **7** did not show any additional signals corresponding to the angolosamine group connected to C8. In addition, the other new peaks at 17 min (**5**) and 18.2 min (**6**) showed the [M + H]^+^ ion signal at *m/z* 403 and *m/z* 361, respectively. One- and two-dimensional NMR analyses identified these compounds to possess agalycone structure (Supplementary Table S6), lacking the aminosugars both at C8 and C10. Compound **5** contained an epoxide in C14, 16 of rubiflavinone C-1 (**3**) and **6** was kidamycinone, an aglycone of kidamycin. Taken together, the accumulation of N,N-dimethylvancosamine glycosylated kidamycinone (**7**) in the ΔKid21 mutant implied that Kid21 was an angolosamine GT for kidamycin. Thus, the N,N-dimethylvancosamine GT reaction takes precedence over the angolosamine GT reaction.

**FIGURE 6:**
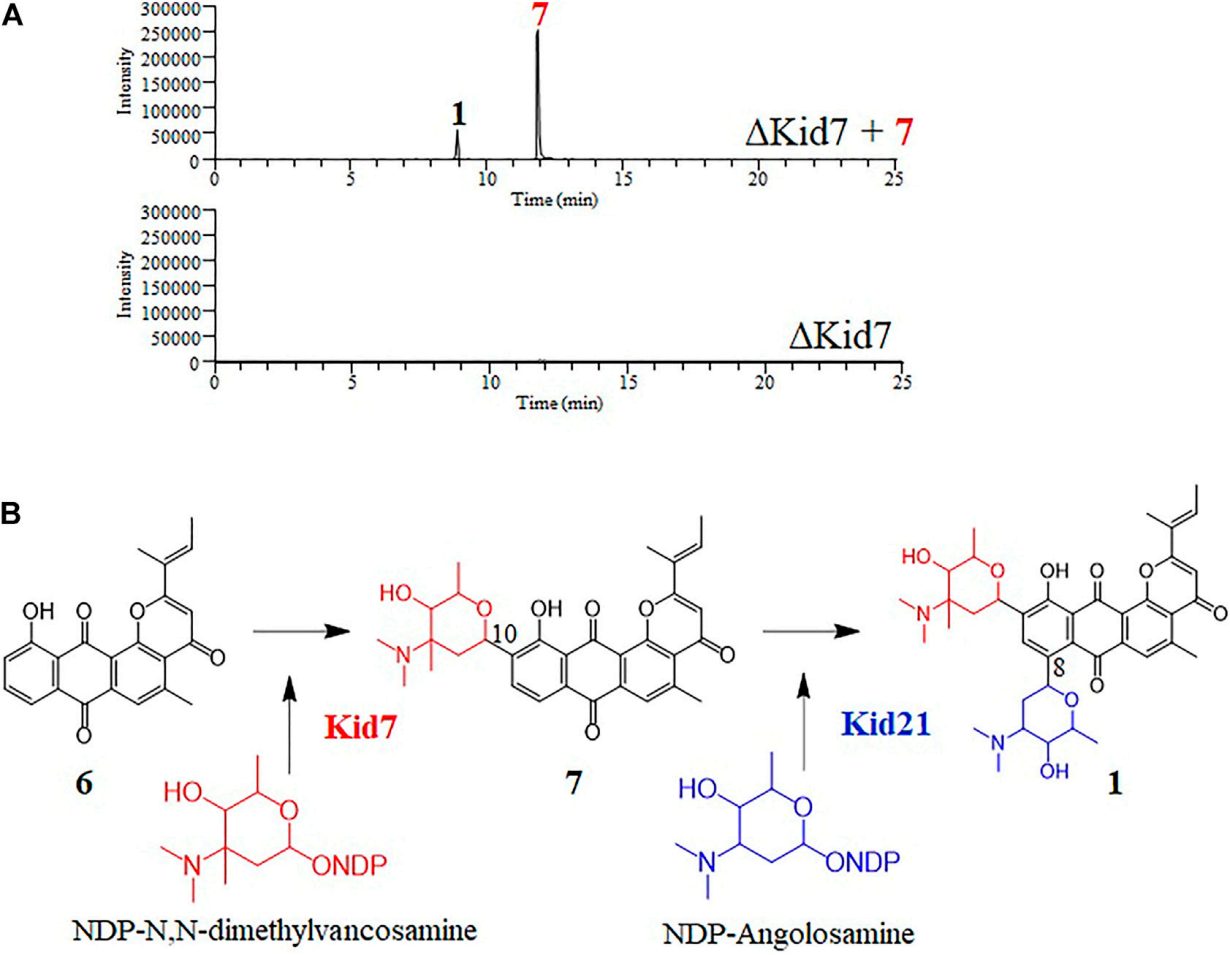
**(A)** Selected LC-MS analysis of the extracts of culture broth obtained from the bioconversion experiment of N,N-dimethylvancosamine glycosylated compound (**7**) by the ΔKid7 mutant. The selected ion chromatograms were pre-set at *m/z* 532 and 689 correspond to the respective molecular ion of compound **7** and kidamycin (**1**), respectively. **(B)** The proposed glycosylation pathway of kidamycin.

Next, we mutated *kid7*, another C-GT gene in *kid* BGC, using the same method as mentioned above. Results showed that the *kid7* mutant produced only aglycone compounds (**3**, **4**, **5**, and kidamycinone) (Figure 5). A new peak at 16.5 min (**4**) was generated at *m/z* = 377 [M + H]^+^, which was identified to be a structure with an epoxide in C14,16 of kidamycinone (Figure 5). As a result, the ΔKid7 mutant produced only aglycone compounds without any attached aminosugar moieties. Therefore, the ΔKid7 mutant produced only aglycone compounds and the ΔKid21 mutant produced *mono*-glycosylated compound. *kid7* gene encodes the C10 N,N-dimethylvancosamine GT and catalyzed the first C-GT reaction in kidamycin biosynthesis. Therefore, Kid21 is expected to be responsible for the second glycosylation step in kidamycin biosynthesis. To verify this, purified N,N-dimethylvancosamine glycosylated compound **7** produced by the ΔKid21 mutant was added to the culture of the ΔKid7 mutant with activated Kid21 C-GT function and angolosamine production. The selected ion peaks were detected on the selected ion monitoring on LC/MS analysis with the pre-set at *m/z* 532 and 689 correspond to the respective molecular ion of compound **7** and kidamycin (**1**), respectively (Figure 6A). The N,N-dimethylvancosamine glycosylated compound **7** was effectively converted to kidamycin in the culture broth of the ΔKid7 mutant. This confirmed that angolosamine glycosylation at C8 is not only the second step in the kidamycin glycosylation pathway, but also that **7** is a substrate of the Kid21 angolosamine C-GT reaction (Figure 6B).

### 3.4 Role of three methyltransferases in aminosugar biosynthesis

The early steps are common with those of aminosugar biosynthesis, which involve NDP-glucose synthase, NDP-glucose-4,6-dehydratase, and NDP-glucose-2,3-dehydratase, all of which were reasonably assigned to the respective gene products of *kid5, kid8*, and *kid26*, respectively, based on significant percent identity (>65%). The Kid22 protein resembles a NDP-deoxyhexose 3-aminotransferase that is involved in the biosynthesis of *Streptomyces* antibiotics containing aminosugars. The late steps for angolosamine biosynthesis, which may be interchangeable, are catalyzed by 4′-keto-reductase and N-methyltransferase. The 4′-keto-reductase genes, med-ORF14 (Cai et al., 2021) and gra-ORF22 (Tornus and Floss, 2001), encode proteins 60% similar to the Kid23 enzyme. Kid4 is highly similar to AORI_1488 (76%), which catalyzes the C-methylation at aminohexose in the vancomycin biosynthetic pathway (van Wageningen et al., 1998; Chen et al., 2000; Xu et al., 2014). Additionally, Kid9 and Kid24 show high identity with med-15 (Kid9: 46% and Kid24: 50%), which catalyze the di-methylation at the amine moiety of angolosamine. (Cai et al., 2021) (Supplementary Table S7)

**FIGURE 7:**
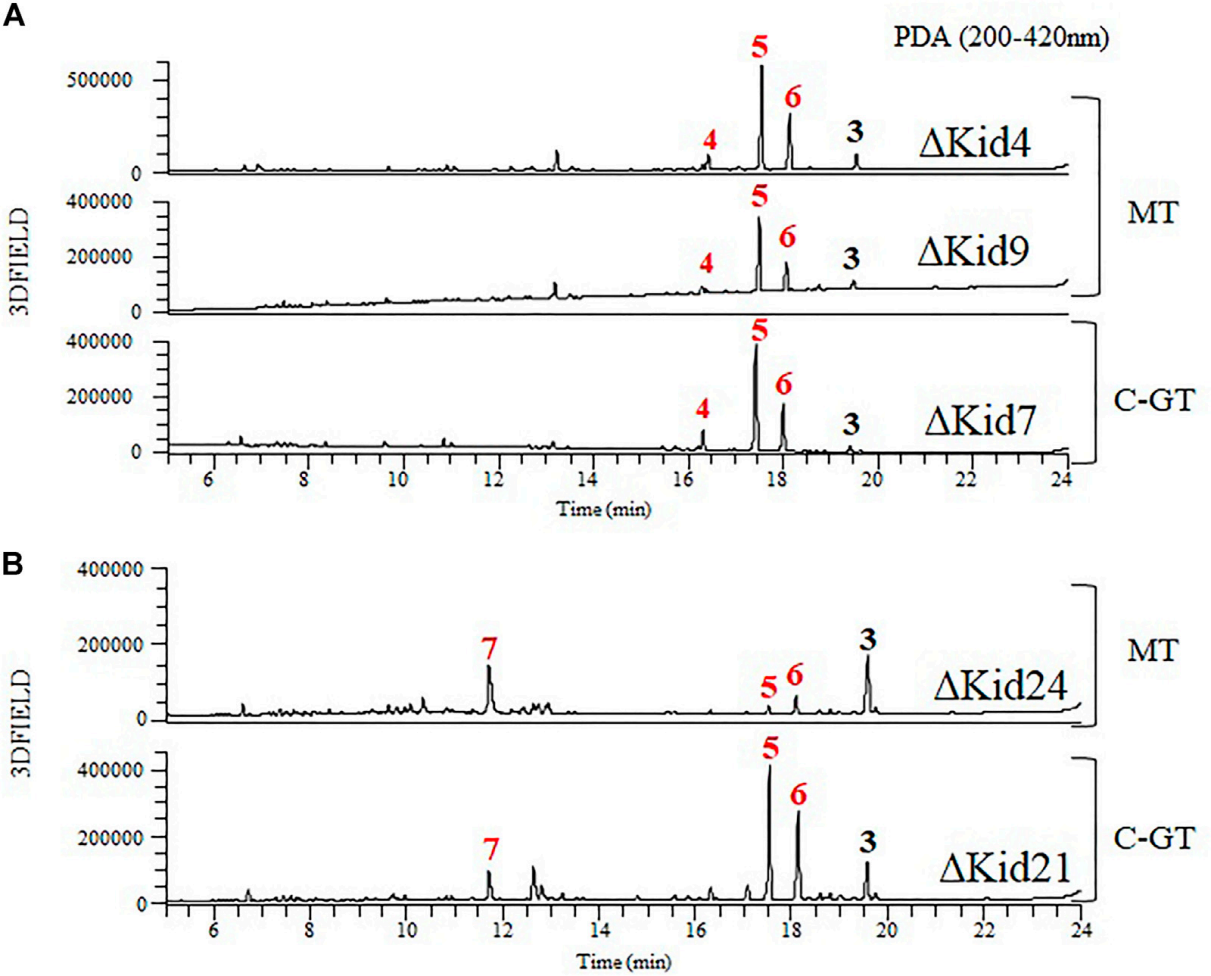
Comparative HPLC analysis of the products of the methyltransferase (MT) mutants and C-GT mutants. **(A)** ΔKid4 (upper), ∆Kid9 (middle), and ∆Kid7 (bottom). **(B)** ΔKid24 (upper) and ∆Kid21 (bottom).

To obtain further insights regarding aminosugar biosynthesis, we inactivated three methyltransferase genes (*kid4*, *kid9,* and *kid24*) and analyzed the function of the methyltransferases. The mutant strains were selected based on kanamycin resistance and genotyping using PCR (Supplementary Figure S1). These mutant strains did not produce kidamycin but yielded new peaks. The peaks produced by the ΔKid4 and ΔKid9 mutants were the same as those of the ΔKid7 C10-GT mutant, and the peaks produced by the ΔKid24 mutant were the same as those of the ΔKid21 C8-GT mutant (Figure 7). This indicated that Kid4 and Kid9 were involved in N,N-dimethylvancosamine biosynthesis, and that Kid24 was involved in the angolosamine biosynthetic pathway. All three methyltransferases (Kid4, Kid9, and Kid24) present in this BGC were involved in the biosynthesis of the aminosugars, N,N-dimethylvancosamine or anglosamine. Interestingly, the two methyltransferase genes (*kid4* and *kid9*) near *kid7* encoded C10 N,N-dimethylvancosamine GT, while *kid24* is a neighbor of the C8 angolosamine GT (*kid21*) gene. Therefore, it is not found in *kid* BGC that corresponds to the methyltransferase of methyl bis-epoxide residue of the hedamycin aglycone. Therefore, the gene corresponds to the methyltransferase of methyl *bis*-epoxide residue of the hedamycin aglycone is not found in *kid* BGC (Bililign et al., 2004).

## 4 Discussion

It is well known that the glycosylation of natural or synthetic compound-based drugs can dramatically influence the pharmacological properties of the parent molecule (Weymouth-Wilson, 1997; Elshahawi et al., 2015). Therefore, the characterization of the biosynthetic pathway of glycoside compounds and the study of the substrates specificity of GTs play an important part in the development of new drugs (Fu et al., 2003; Salas and Méndez, 2007; Singh et al., 2012). In particular, the development of new derivatives using GT enzymes with relatively broad substrate promiscuity is being actively carried out (Luzhetskyy et al., 2008; Pandey and Sohng, 2016; Xie et al., 2020; Goel et al., 2021). However, most of these approaches are used with O-GTs, only a small number of C-GTs have been found and are limited in their use.

C-GTs catalyze the transfer of activated sugar moieties to the carbon atoms in substituted aromatic rings of receptor molecules and generally show high selectivity for receptors and sugar moieties. Because of the regio- and stereospecifically attached sugar moieties, C-GTs also play important roles in biological activities and in the biosynthesis of pharmaceutically significant natural products (Kren and Martínková, 2001; Elshahawi et al., 2015; Putkaradze et al., 2021). As is evident from the recent development of various synthetic derivatives for pluramycin family compounds, modification in the sugar moieties is an important factor determining their pharmacological activities. However, compared to the studies on synthetic derivatives, those on biosynthetic pathways are rare, with the only reports being on the discovery of the 2 GTs, HedJ and HedL, in hedamycin BGC (Bililign et al., 2004; Das and Khosla, 2009).

This is the first study to show the precise glycolytic function of Kid7 and Kid21, 2 GTs involved in kidamycin biosynthesis, and the biosynthetic order of each C-GT using gene disruption and bioconversion experiments. Both Kid7 and Kid21 showed significant homology with angolosamine C-GT of medermycin (Med-8), although the identity of Kid21 was slightly more than that of Kid7. Owing to these slight differences, Kid21 was classified as angolosamine C-GT, while Kid7 was classified as N,N-dimethylvancosamine C-GT. Thus, both C-GTs showed strong selectivity for sugar moieties and regiospecifically attached sugar moieties to the different carbon atoms in the angucycline ring of kidamycin. This study provides a basis how enzymatically generates unusual sugars attached angucycline compounds and has paved the way for alteration of these glycosylated compounds via pathway or enzyme engineering (Blanchard and Thorson, 2006; Zhao and Liu, 2010; He et al., 2019). *Streptomyces* sp. W2061, a kidamycin producer, also accumulated rubiflavinone C-1, which harbors an aglycone with a hexenoate residue, unlike the 2-methylbutenoyl residue of kidamycin, under the same culture condition. The glycosylated derivatives of rubiflavinone are also detected in small amounts in the same culture by molecular networking searching (data not shown). This indicated that among the two different aglycones of varying chain lengths (kidamycinone and rubiflavinone), the 2 GTs have considerable specificity for 2-methylbutenoyl of kidamycinone, although they are produced by a shared aglycone biosynthetic system that possibly uses two different precursors. Therefore, replacement with C-GTs of hedamycin, which mainly produce aglycone with hexenoate residue, will provide new insights regarding the substrate specificity of C-GT.

Additionally, three methyltransferase genes showed homology with the genes previously reported to be involved in amniosugar biosynthesis. Kid4 and Kid9 are involved in N,N-dimethylvancosamine biosynthesis, while Kid24 is involved in the angolosamine biosynthetic pathway. Interestingly, these genes were located near the C-GT genes, and each of these methyltransferases used biosynthetic aminosugars as substrates. *kid4* and *kid9* are adjacent to the N,N-dimethylvancosamine GT (*kid7*), and *kid21* is adjacent to *kid24*. These two loci are separated by the PKS genes. However, genes involved in the early biosynthesis of aminohexose, glucose-1-phosphate thymidyl transferase (*kid5*), NDP-4-keto-6-deoxyhexose 3,5-epimerase (*kid6*), TDP-glucose 4,6-dehydratase (*kid28*), and NDP-deoxyhexose 3-aminotransferase (*kid20*) are scattered on both sides of the glycosylation gene locus.

In conclusion, we demonstrated that the two C-GTs, Kid7 and Kid21, in *Streptomyces* sp. are involved in sequential glycosylation in kidamycin biosynthesis. Kid7 first attached N,N-dimethylvancosamine to the C10 position of aglycone, following which Kid21 transferred an anglosamine moiety to C8 of the C10-glycosylated kidamycinone. This is the first report to reveal the sequential biosynthetic steps of the C-glycosylated amino-deoxyhexoses of pluramycin family angucycline compounds. Additionally, all the three methyltransferases (Kid4, Kid9, and Kid24) present in the kidamycin BGC were involved in the biosynthesis of these amino sugars, and were not related to methylation of the side chain of aglycone, as predicted in the production of methyl bis-epoxide residue of hedamycin. The nine aglycones and one mono-glycoside compound produced in these genetically modified strains will provide new substrates for bioconversion experiments with other C-GTs or for organic synthesis, which will assist in developing pluramycin-related natural/non-natural compounds.

## Data Availability

The original contributions presented in the study are included in the article/Supplementary Materials, further inquiries can be directed to the corresponding authors.

## Abbreviations

BGC, biosynthetic gene cluster; GT, glycosyl transferase; PKS, polyketide synthase; LC-MS, liquid chromatography-mass spectrometry.
