# Supplementary material for: Elucidation of the di-c-glycosylation steps during biosynthesis of the antitumor antibiotic, kidamycin
Source: Front Bioeng Biotechnol. 2022 Aug 25;10:985696. doi: 10.3389/fbioe.2022.985696 (PMC9452638; doi:10.3389/fbioe.2022.985696)
Supplement: Supplementary file 1 [file DataSheet1.DOCX]

Supplementary Materials

Elucidation of the Di-C-glycosylation Steps during Biosynthesis of the Antitumor Antibiotic, Kidamycin

Kyung Taek Heo ^1,2^, Byeongsan Lee^1^, Jae-Hyuk Jang^1,2,*^, Young-Soo Hong ^1,2,*^

^1^Chemical Biology Research Center, Korea Research Institute of Bioscience and Biotechnology, 30 Yeongudanji-ro, Ochang-eup, CheongJu-si, Chungbuk 28116, Republic of Korea

^2^Department of Bio-Molecular Science, KRIBB School of Bioscience, University of Science and Technology(UST), Daejeon 34141, Republic of Korea

*** Correspondence:**

Jae-Hyuk Jang (jangjh@kribb.re.kr)

Young-Soo Hong (hongsoo@kribb.re.kr)

***Supplementary Tables***

**Supplementary Table 1.** Plasmids and strains used in this study.


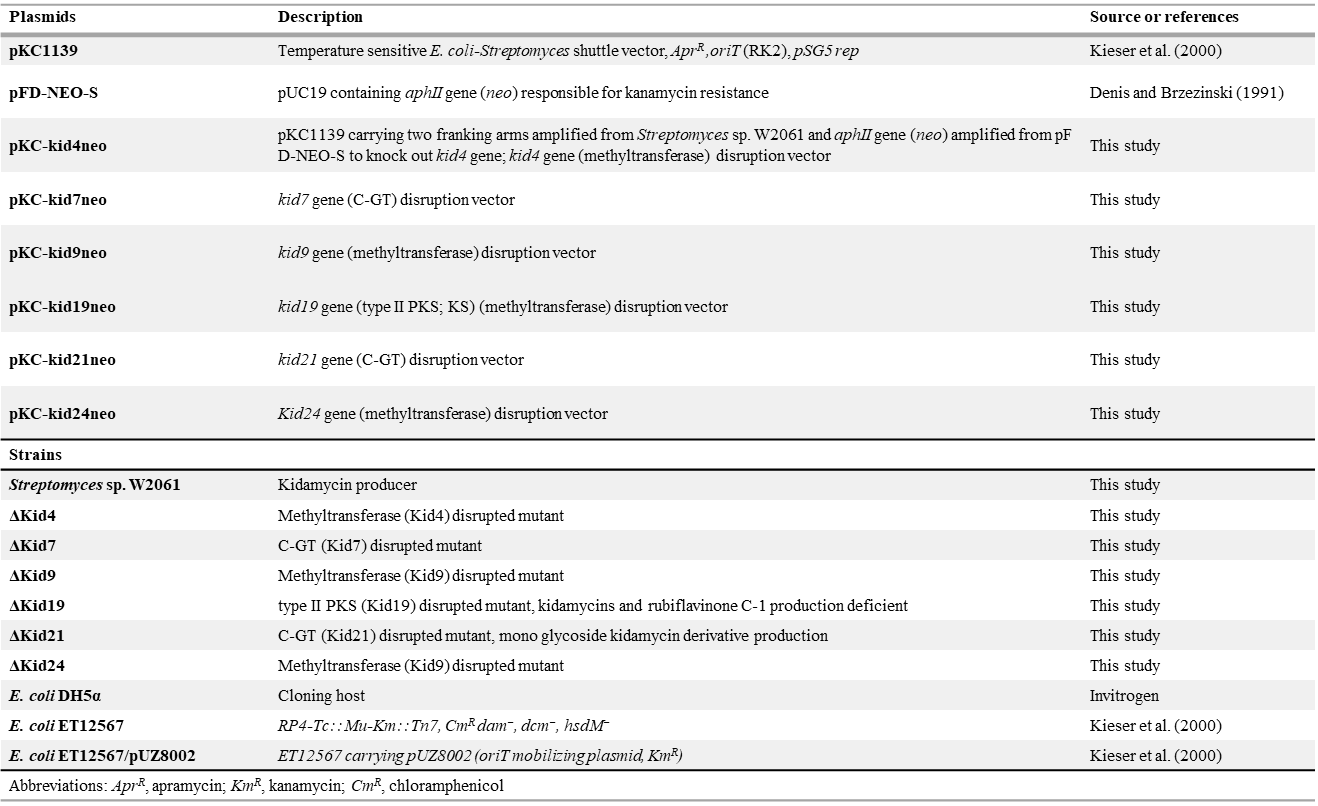


**Supplementary Table 2.** Primers used in this study.


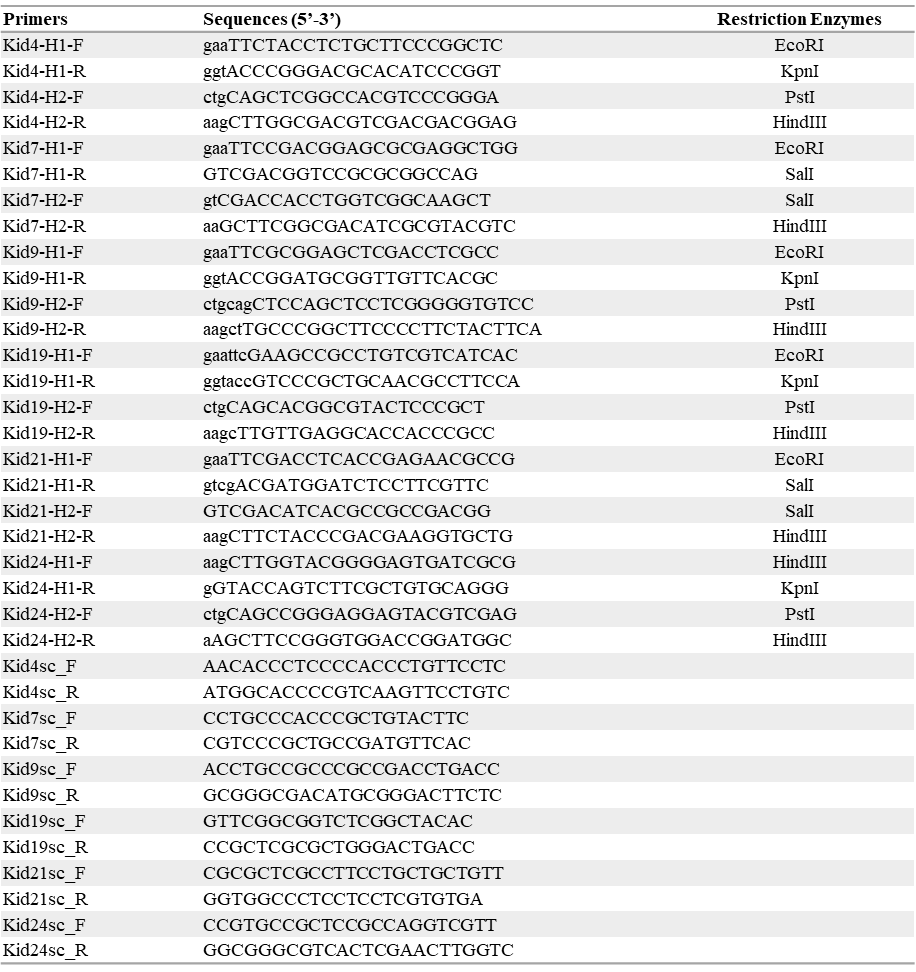


**Supplementary Table 3**. Deduced function of the kidamycin ORFs.


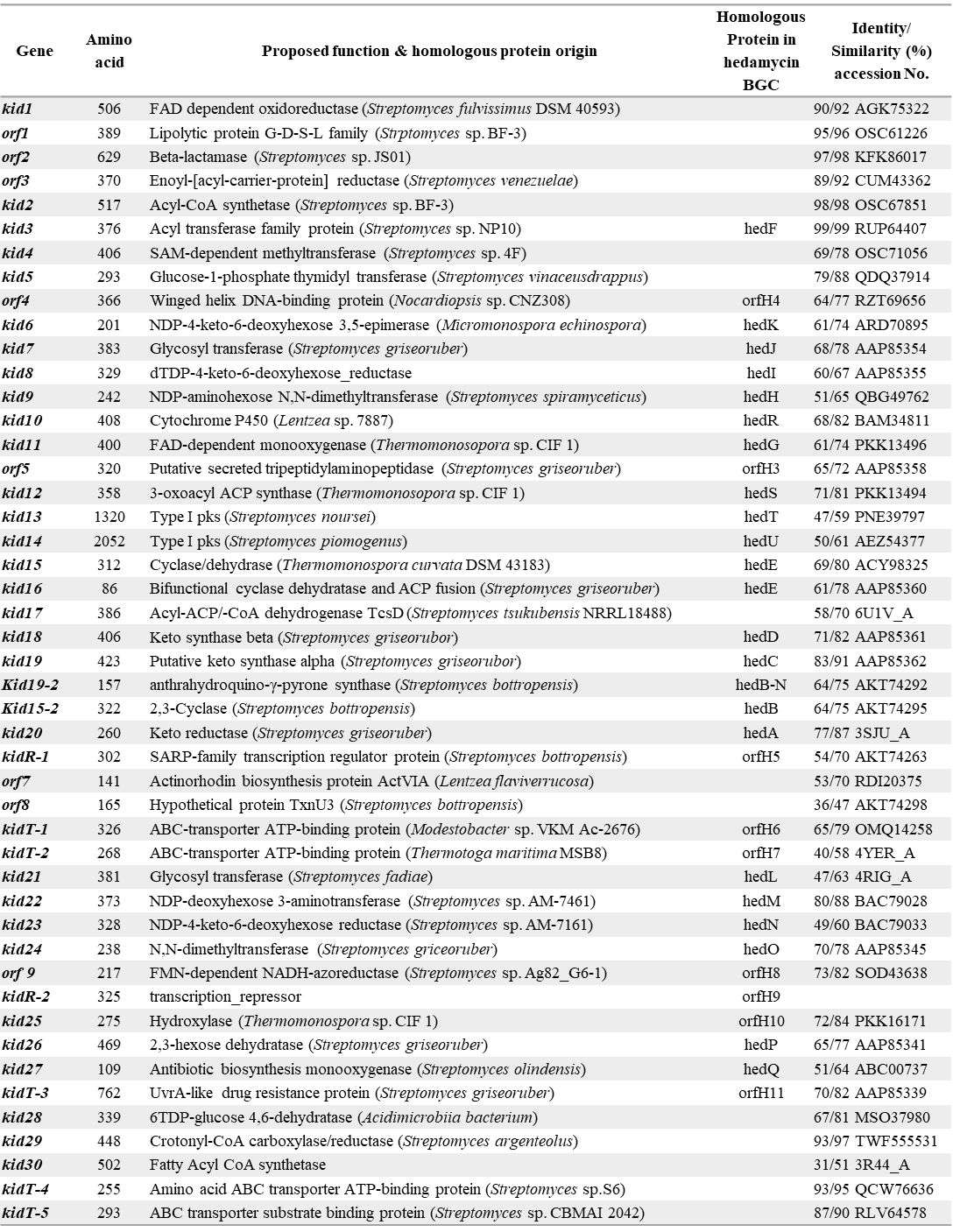


**Supplementary Table 4**. Percentage of identity between the amino acid sequences of kidamycin C-GTs and those of other microbial C-GTs. HedJ and HedL are C-GTs from hedamycin BGC, Med8 is angolosamine C-GT from medermycin BGC, and UrdGT2 is olivose C-GT from urdamycin BGC.

|  | Protein sequence identity (%) | | | | |
| --- | --- | --- | --- | --- | --- |
| Enzyme | HedL | Kid7 | Kid21 | Med8 | UrdGT2 |
| HedJ | 35.4 | 67.5 | 33.2 | 35.6 | 32.1 |
| HedL |  | 34.5 | 71.8 | 47.6 | 43.8 |
| Kid7 |  |  | 33.7 | 37.8 | 31.8 |
| Kid21 |  |  |  | 47.3 | 41.6 |
| Med8 |  |  |  |  | 49.9 |

**Supplementary Table 5**. ^1^H and ^13^C NMR spectroscopic data NMR data of compound **7** and kidamycin (**1**).


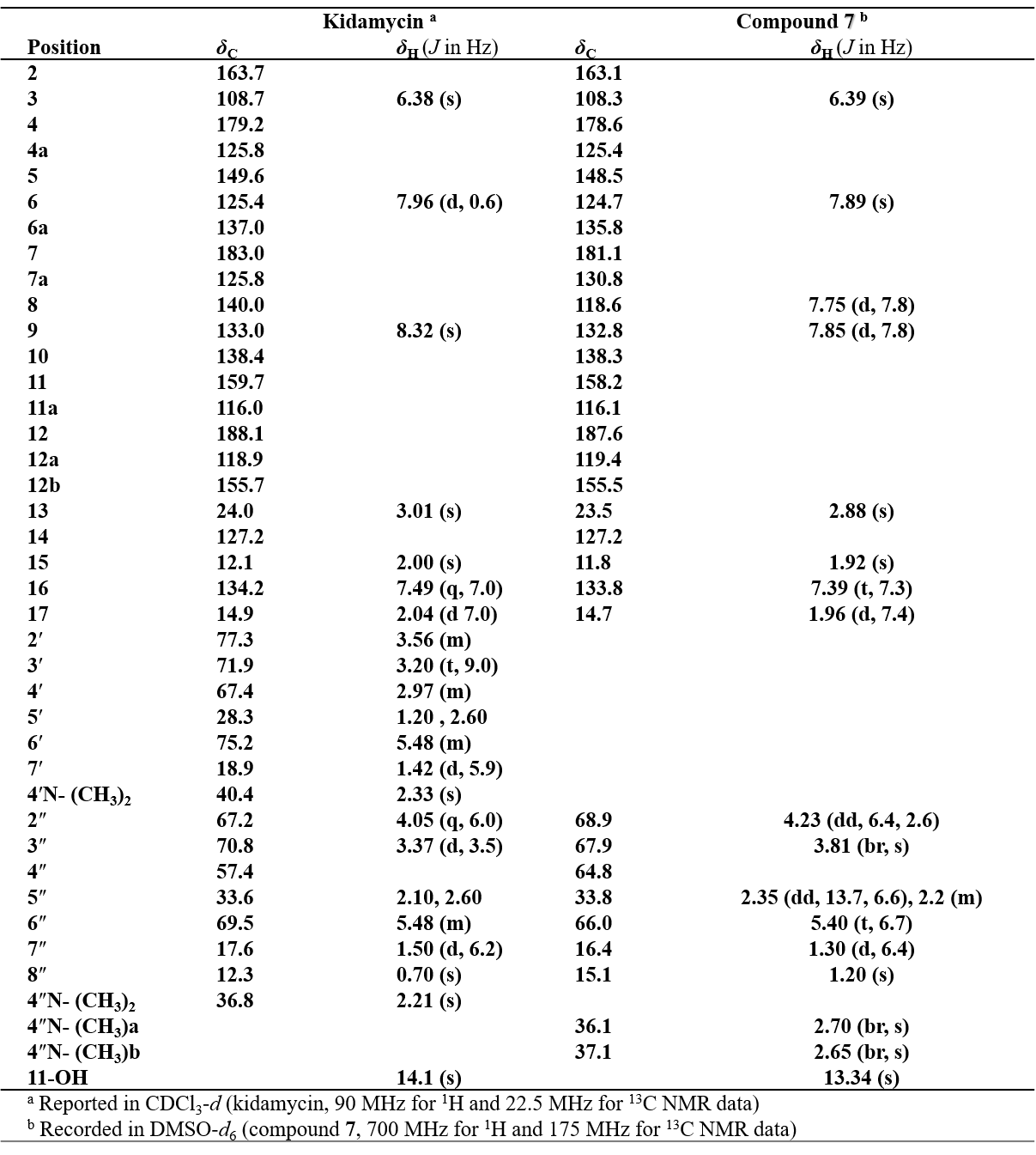


**Supplementary Table 6**. ^1^H (700 MHz) and ^13^C (175 MHz) NMR data for compounds **3-6** in CDCl_3_-*d*.

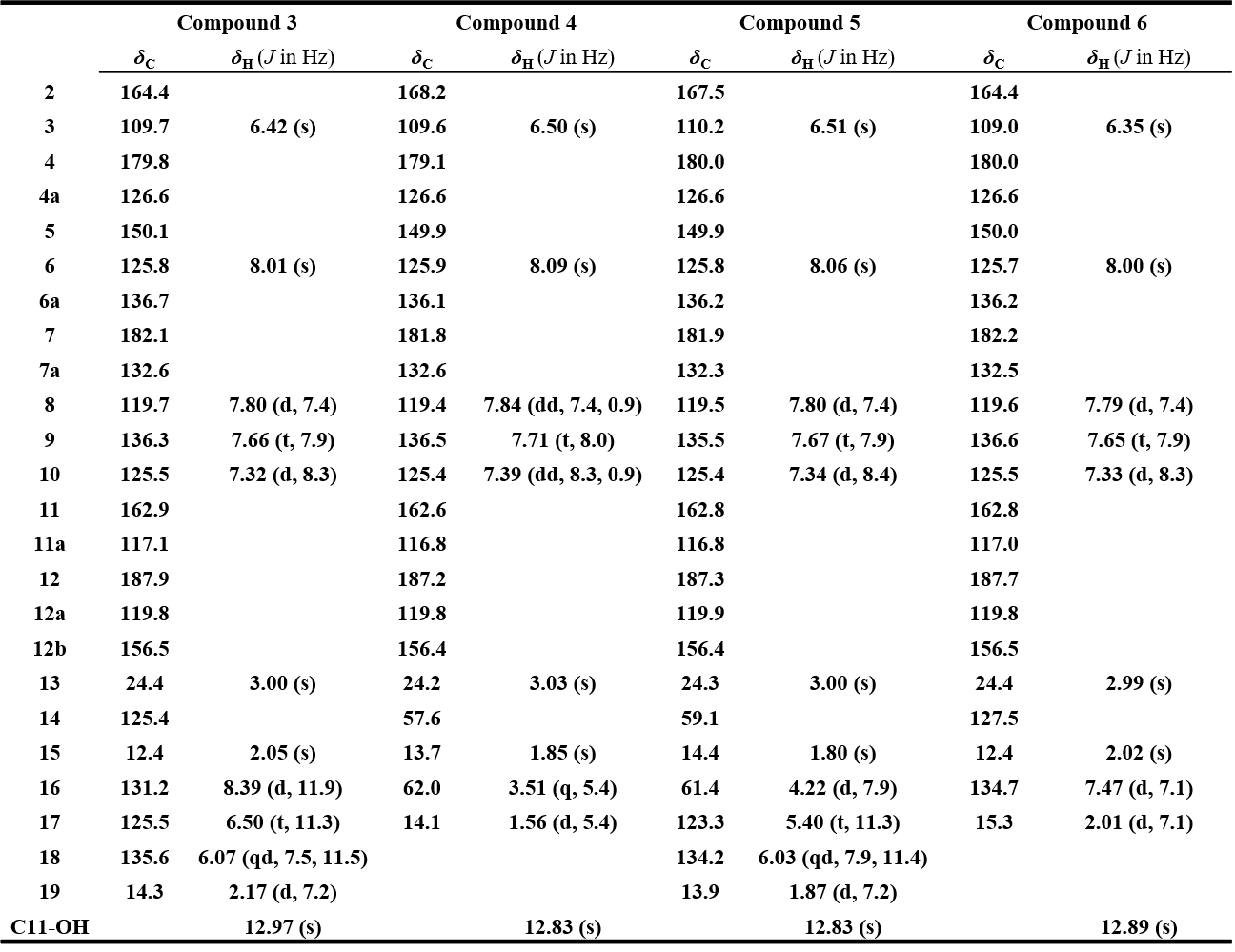


**Supplementary Table 7**. Deduced function of genes involved in aminosugar biosynthetic pathway in the kidamycin BGC and homologous genes in medermycin (Med) and vancomycin (Van) BGC.


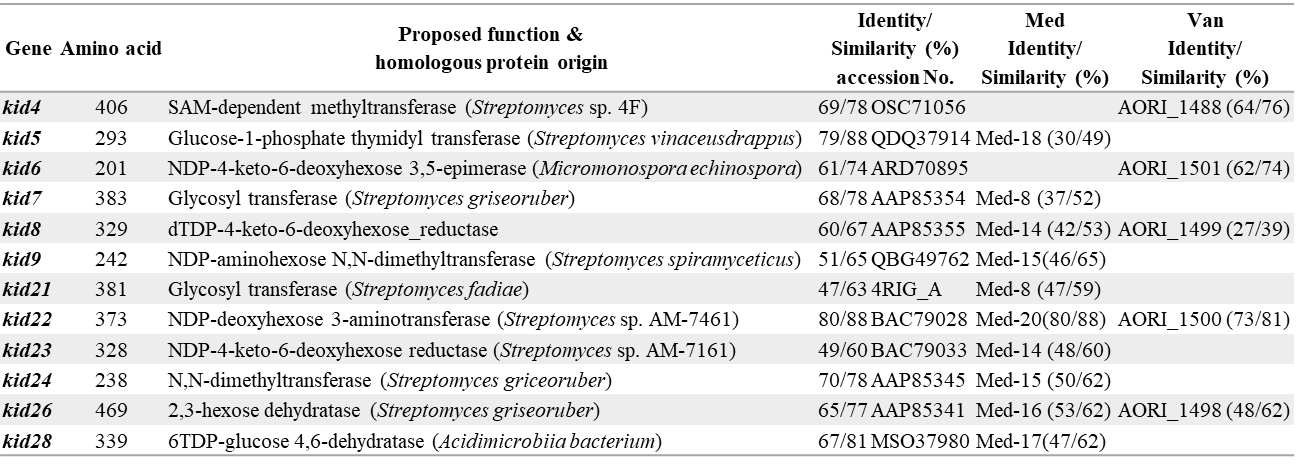


***Supplementary figures***

**Supplementary Figure 1.** Strategies for gene disruption. The mutant resulted from a double crossover event to produce a kanamycin-resistant strain in which the target genes were disrupted (i). Confirmation of insertional gene inactivation using PCR and the total genomic DNA of each mutant as the template (ii). The primers (Supplementary Table 2) used to amplify the desired DNA fragments are indicated by solid arrows. M, 1 kb ladder; 1, wild type; 2, mutants. (A) ∆Kid21, (B) ∆Kid7, (C)∆Kid4, (D) ∆Kid9, (E) ∆Kid24


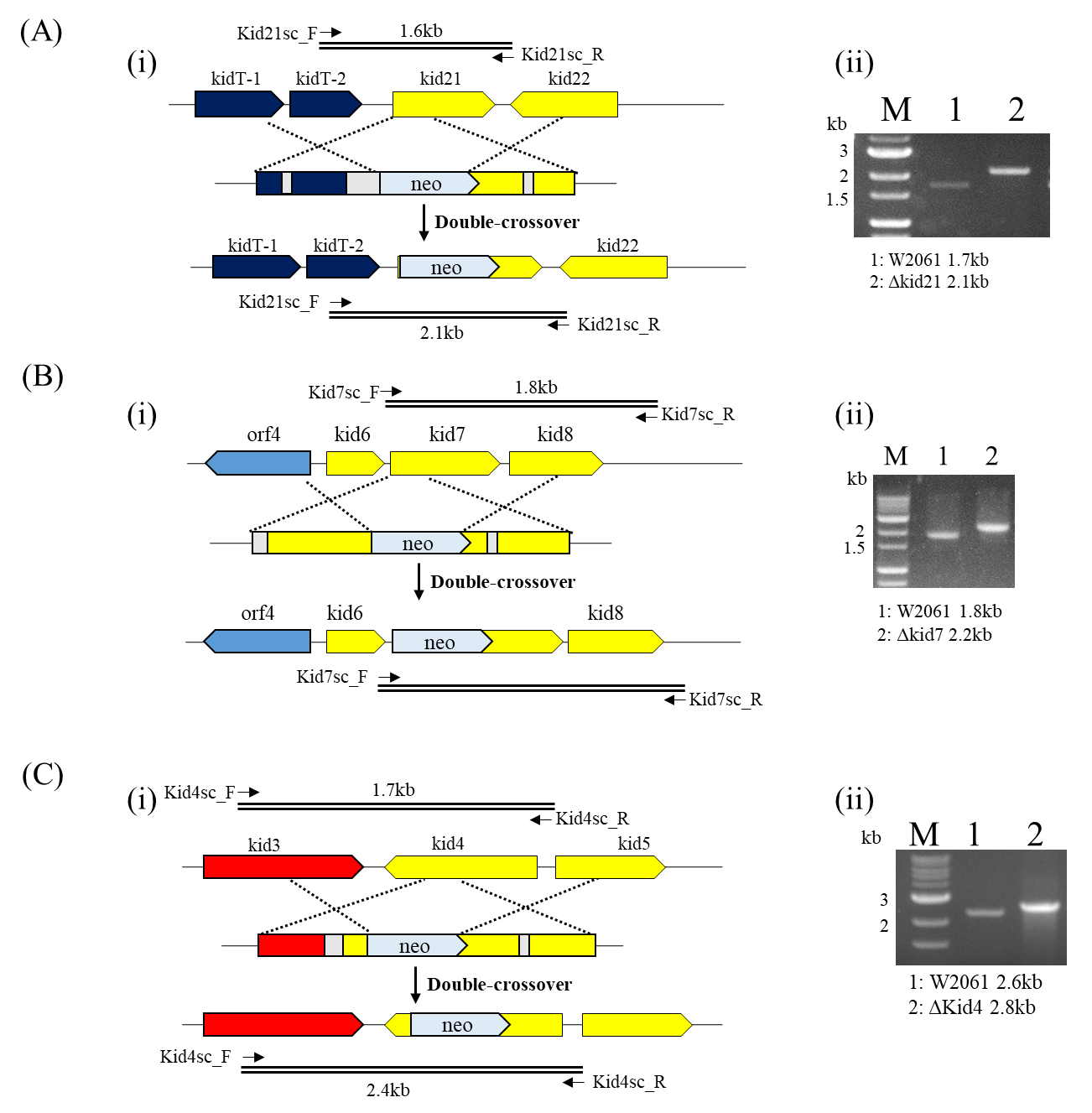


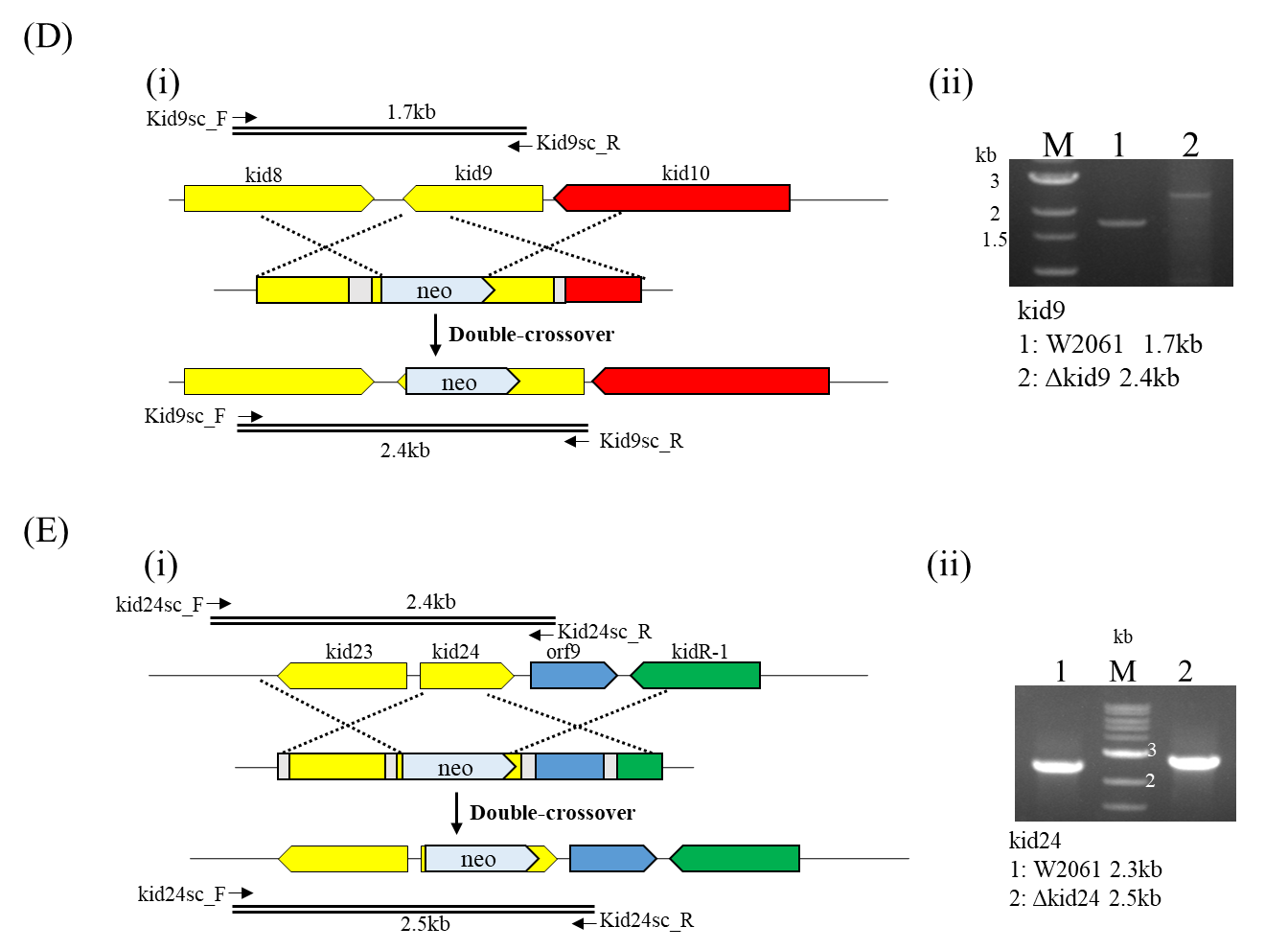


**Supplementary Figure 2.** Proposed biosynthetic pathway for kidamycin production.


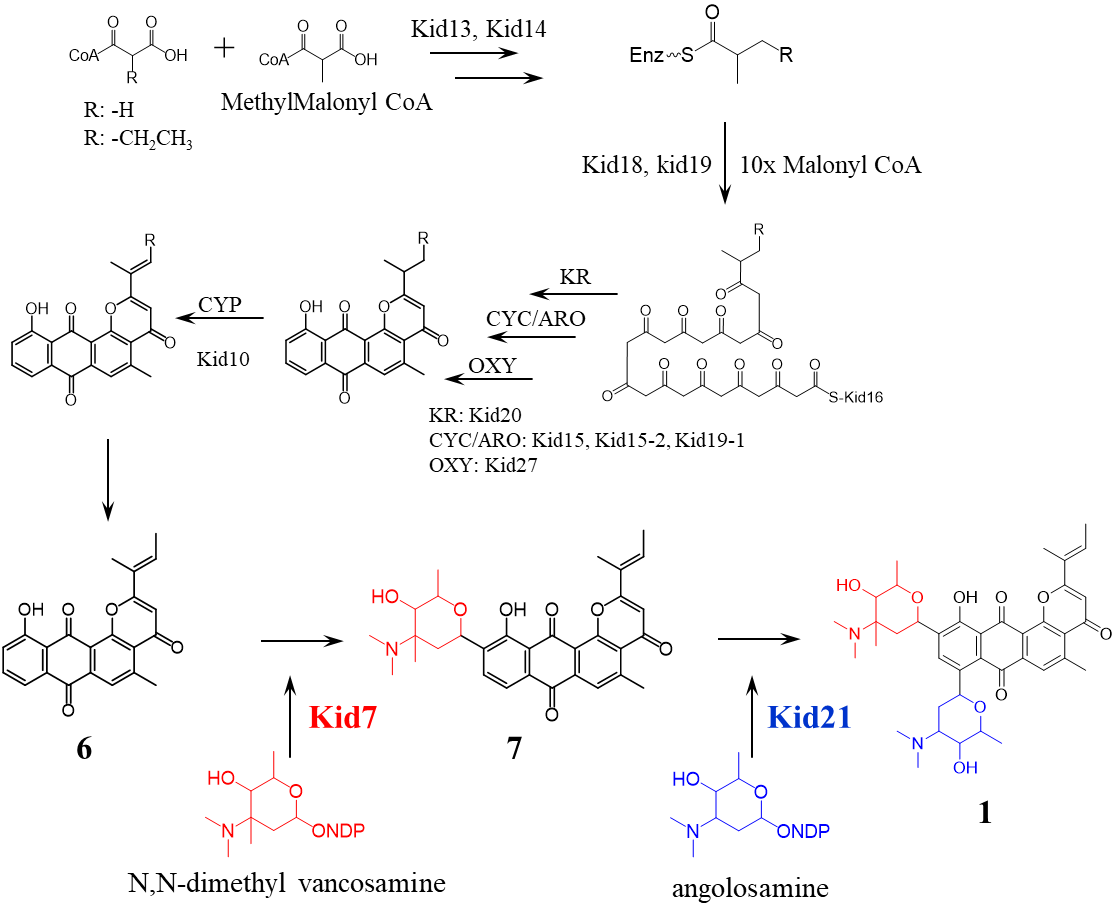


**Supplementary Figure 3**. LC-ESI–MS (upper) and MS/MS (bottom) profile of mono glycosylated compound **7**. (Upper) Detection of precursor ion at *m/z* 532 [M+H]^+^. (Bottom) MS/MS fragmentation of precursor ion obtained in the LTQ trap. The mass region of the spectrum was magnified to highlight neutral losses in the N,N-dimethylvancosamine moiety.

**
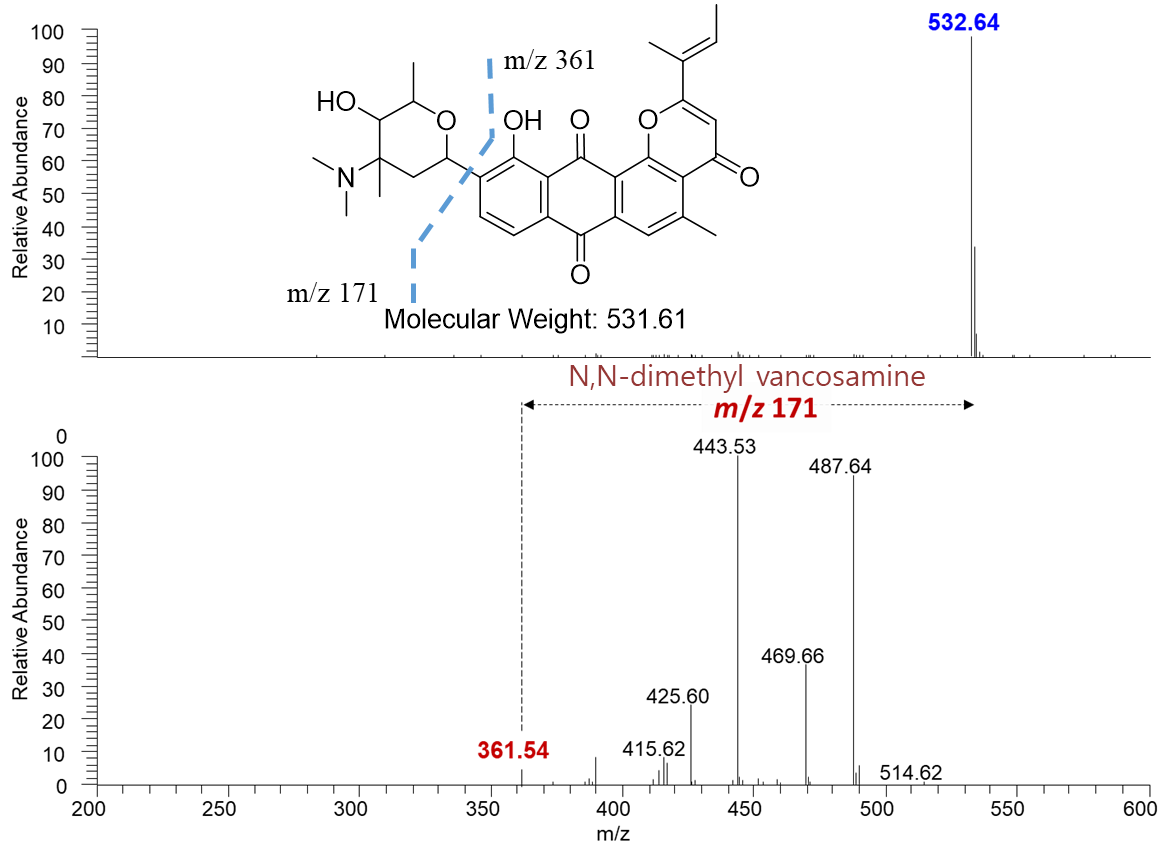
**

**Supplementary Figure 4**. Key 2D NMR correlation of compound **7**.

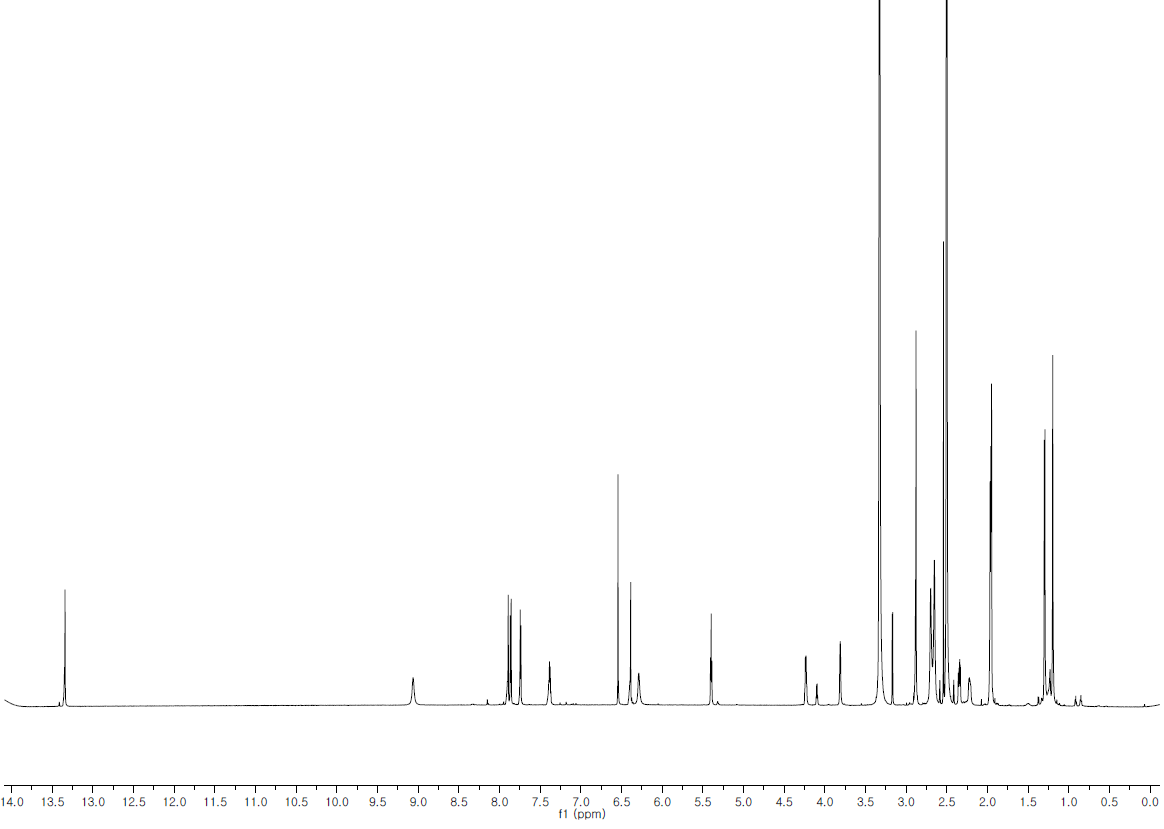


**Supplementary Figure 5.** ^1^H NMR spectrum (700 MHz) of compound (**7**) in DMSO-*d*_6_


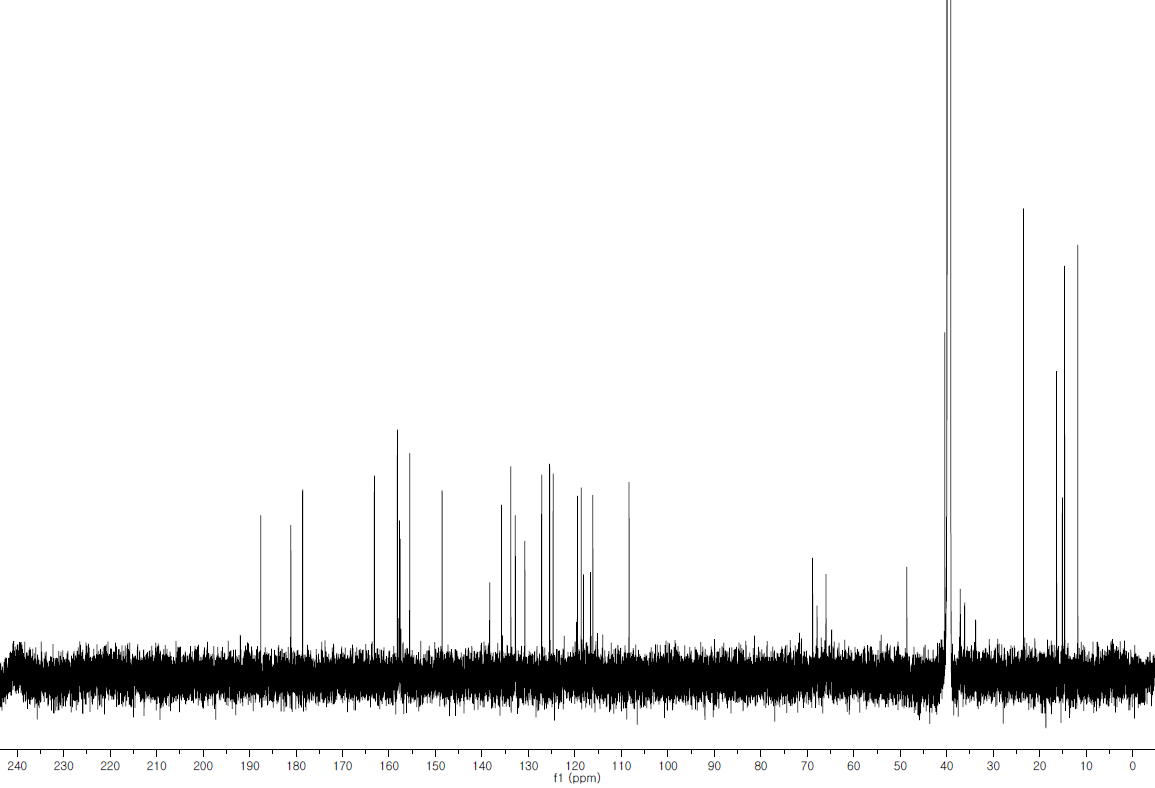


**Supplementary Figure 6.** ^13^C NMR spectrum (175 MHz) of compound (**7**) in DMSO-*d*_6_


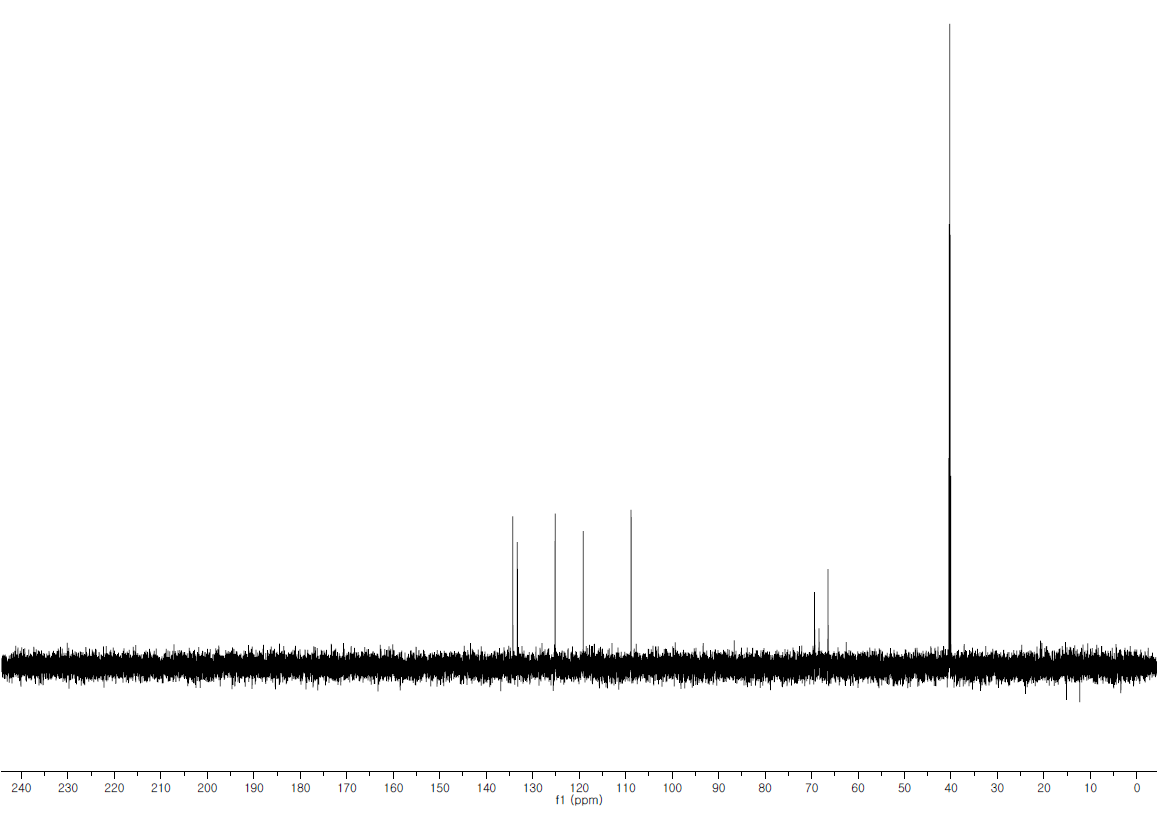


**Supplementary Figure 7.** DEPT90 spectrum of compound (**7**) in DMSO-*d*_6_


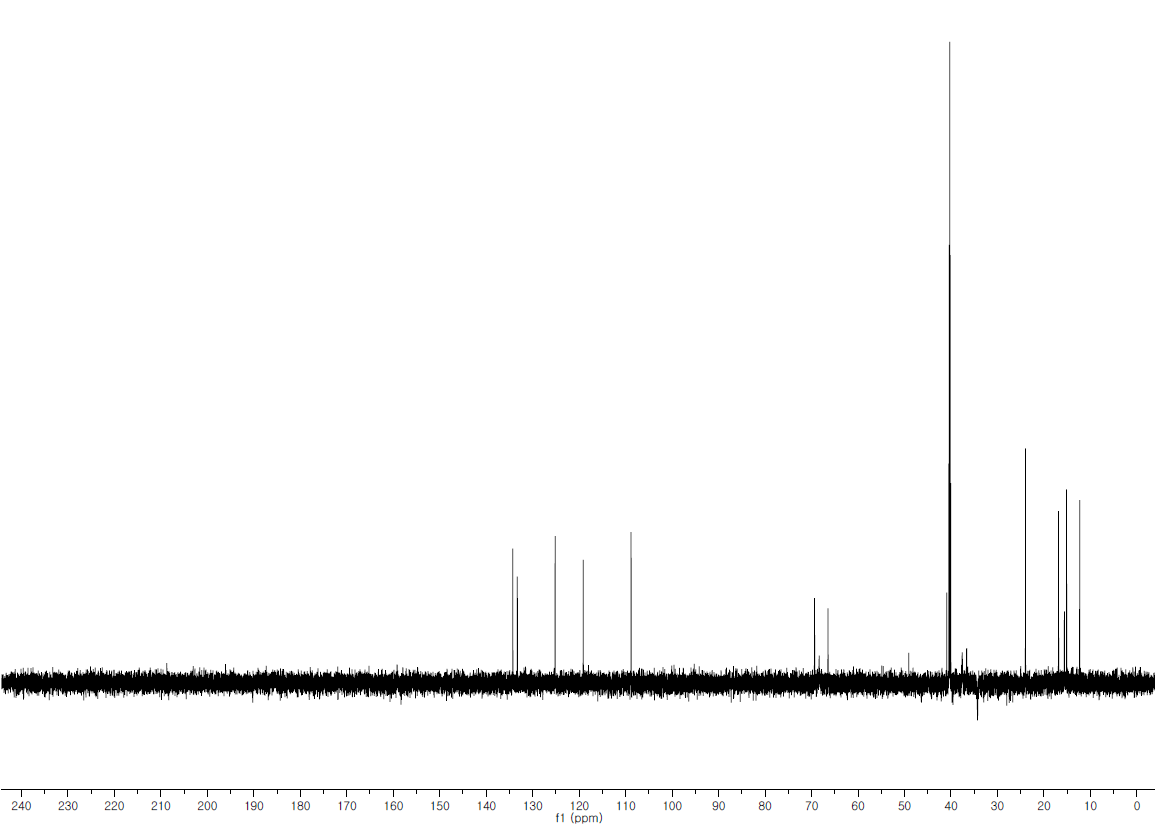


**Supplementary Figure 8.** DEPT135 spectrum of compound (**7**) in DMSO-*d*_6_


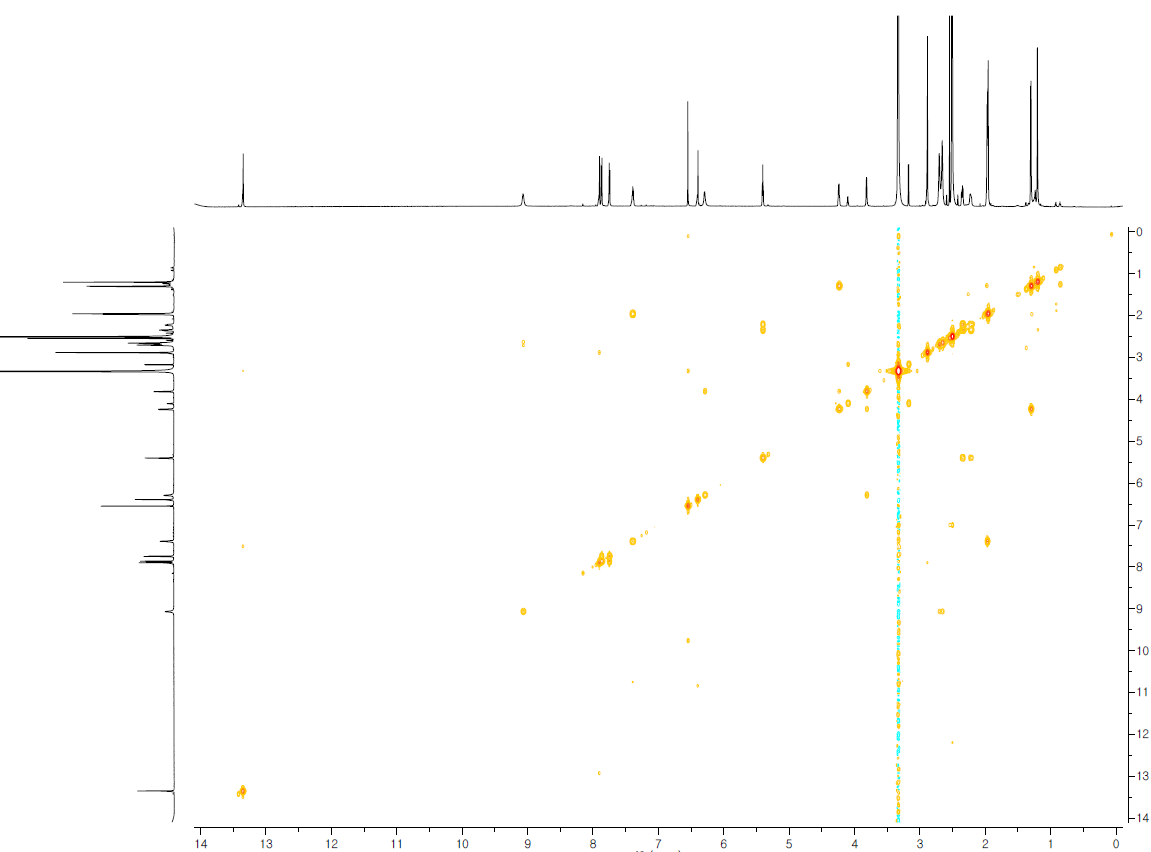


**Supplementary Figure 9.** COSY spectrum of compound (**7**) in DMSO-*d*_6_


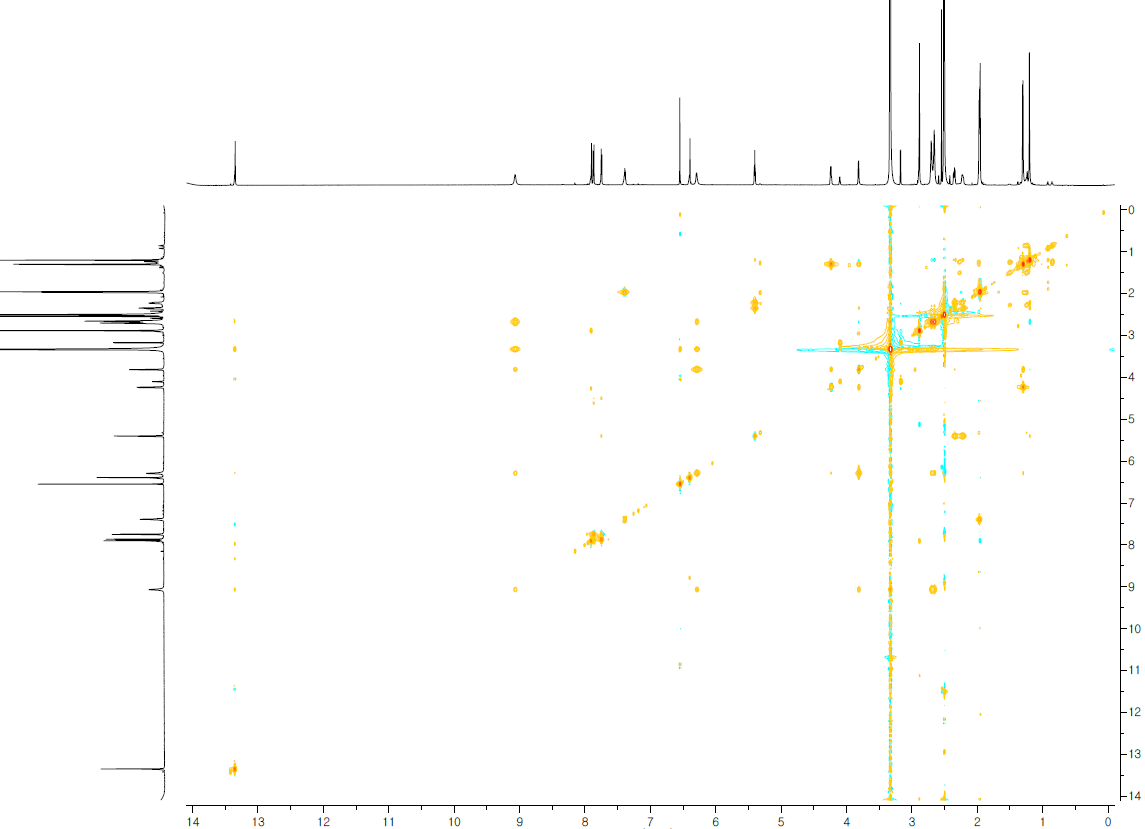


**Supplementary Figure 10.** TOCSY spectrum of compound (**7**) in DMSO-*d*_6_


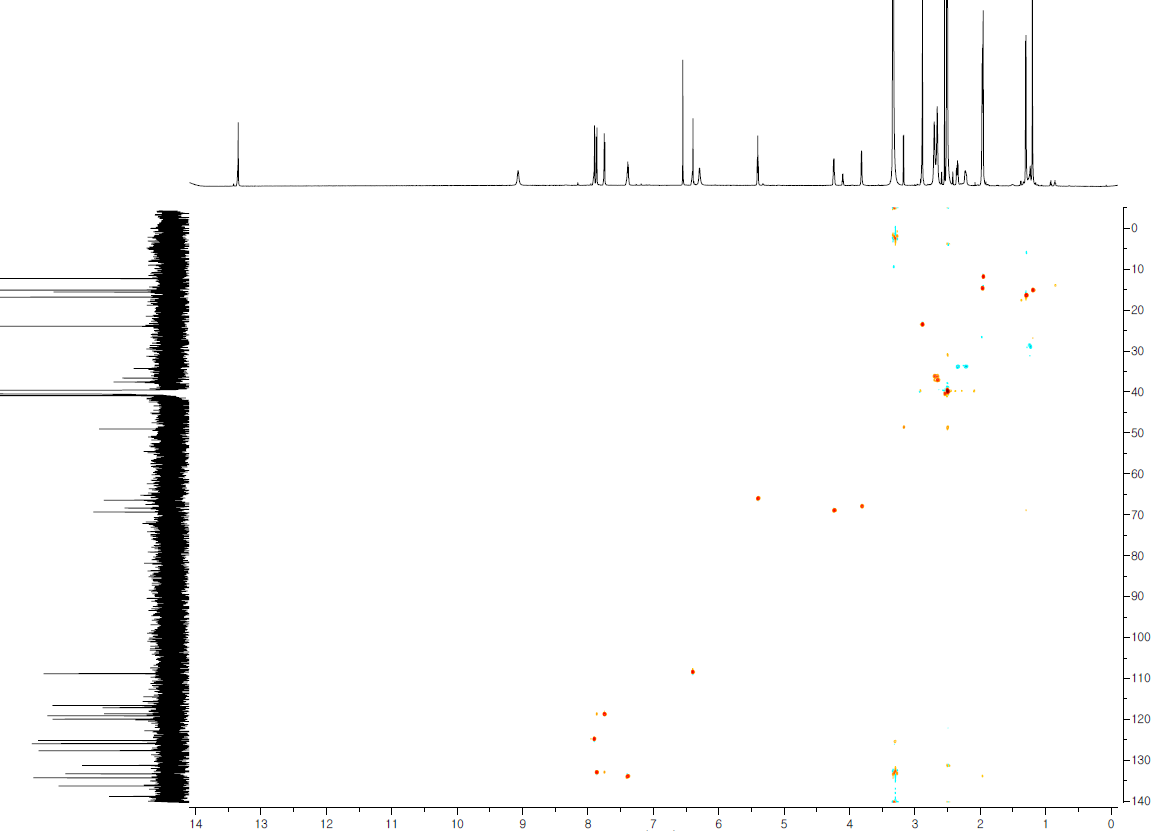


**Supplementary Figure 11.** HSQC-DEPT spectrum of compound (**7**) in DMSO-*d*_6_


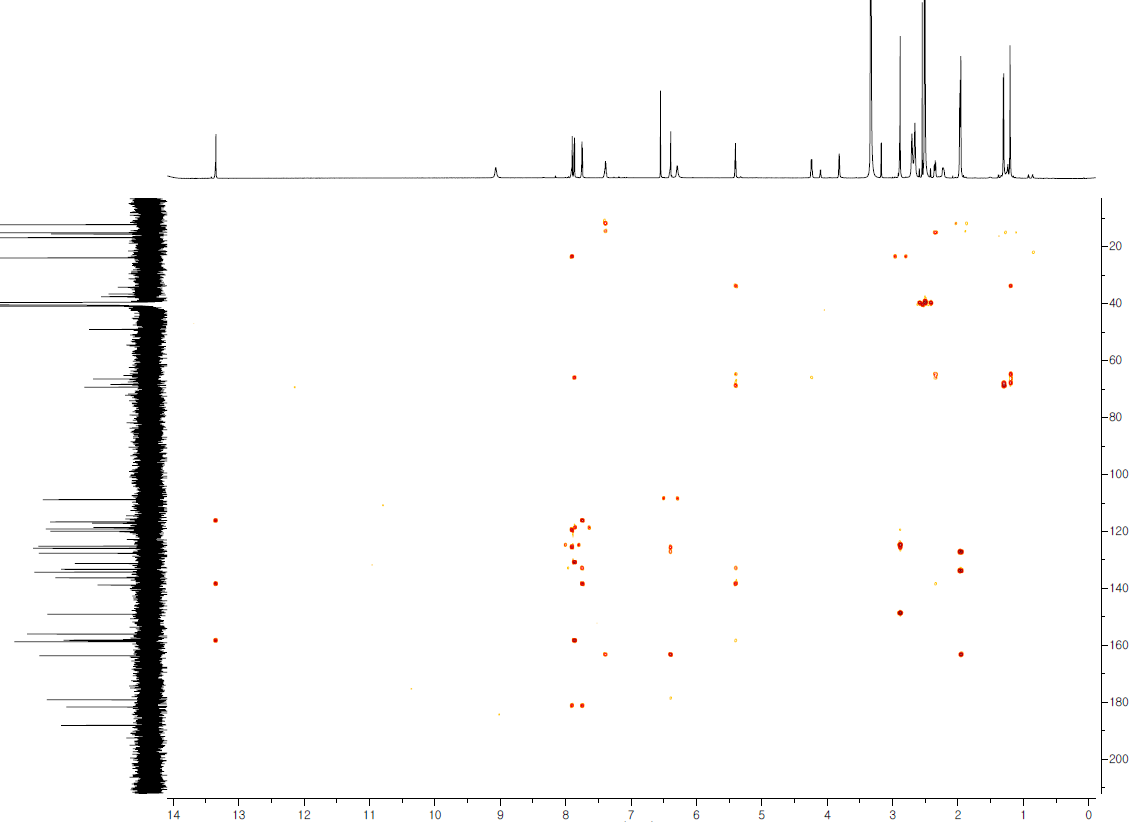


**Supplementary Figure 12.** HMBC spectrum of compound (**7**) in DMSO-*d*_6_

> Kid4; methyltransferase

MTSCRICQGVVQEFFDFGRQPLSDAFRRPEDSSEEFFYRLAVGVCEQCTMVQLMEEVPRERMFRADYPYHSSMSSVMRDHFTRTAHQLLAAGGQGEDPFVVEIGCNDGVMLKTVAEQGVRHLGVDPSRDVAELAAGKGVRVQIDFFQESTATAIRETDGPADVIYAANTICHIPYLDSVFRGVDALLAPDGIFVFEDPYLGDIVERTSFDQIYDEHFYLFSASSVRATARRFGFELVDVERLPVHGGEVRYTIGREGRREPAAAVDALIAEERKRQIVDITILENFAVEVKRNCEALTTLLAQLHEQGHTVAAYGATAKSATVANYCDIGPDLVPAVFDSTAAKQGRLTPGTHIPVRPMEEFSAPYPDYALLFAWNHADEIIAKEQEFRARGGKWIVYVPEVKII

> Kid7; glycosyltransferase

MKILVATGPAHPLYFPVVPLAWALRAAGHEVLVTVPESFESTVSGSGLAMTPVHGPLDMGEVMALDREGRPVRVPDNDADMAAGVGAGFGRLAARTVDATVELVGRWRPDLVITDSYSFAAPAIAAGIHGVPWVKHVVGPGDLPVADAVERELAPELERLGLDKLPPPALVLDNCPPSLGDPAPGAQPVRYVPFGEPGAVPGWVHEPRTRPRLLVTLGSVQPQIGGIPMLGQMIQALASLDAELVVAVADHLVGKLGTLPDKVVAAGWISLTSVLPGCDAAVHHGGPGTMMACLAQGIPQVVVPGRGKPLEAIGRLADLGAVRHLPPADLTPQSLLDSCRTLLEDTGHAKRAMEVREEIARQPSPGAVVPVLEDLVLRYRTA

>Kid9; N,N-dimethyltransferase

MAEEVYGSGLAEIYELIYAGRGKDYPAESAEVAAHVRARRPDAGTLLDVACGTGGHMAFLRETFDVVEGLELSEHMIVKAGESMPGLPVHAGDMRDFSLERSYDAVICMFSSIGYMDTPEELEAALSGMARHLTPGGVIVLEPWYFPDAFLPGYIAEDLVRSDGRVTVRISHSTREGDRVPIVVHYLDALKDGGIRHFTDVHRMRLFTREAYERAFEEAGCSVEYIRTDRFGCGLFVGVLK

> Kid19; Type II PKS ketosynthase alpha

MARRAVITGIGVVAPSGIGREPFWQLLTSGRTATRTITQFDASEHRCRVAAECDFDPLALGLTRQQARRTDRVSQLAMVAAGEALADSGLELDGELAERTGVAIGSAVGGTTTMEREYAVLSNGGKDWVVDPSFAPPHLYDYFLPSSIASEVALTTGALGPAAVISTGCTSGIDSVGYAAELVRDGSADVMITGATDAPISPITLTCFDVIKATSARNDDPATACRPFDRTRTGLILGEGSAVLVLEEYEHARRRGATIYAEVAGYASRCNAFHMTGLKADGKEMSEAIDLALGDARLDPTAIDYVNAHGSGTKQNDRHETAAVKLSLGDHAYRTPMSGIKASVGHSLGAIGSIEIAACALSMRHGVVVPTANLHEADPECDLDYVPNTARERELGAVLTVGSGFGGFQSAMVLKNLERSAS

>Kid21; glycosyltransferase

VRFLFVVGGGSAPVHASVPLAWAARAAGHEVLVASPEENLDLVTGLGLPARGVTDLGMGDAMLKDRQGNWLPMPTSESTEMDFAGRGFARLSAASYRGTEELAEAWRPDVVIGGEYNHAAPLIAHKFSIPLVSHTWAIYDRTDIDWQGATDELKPELAAFGLDAIPEPALFVDITPPTVRPGHAEPAQPMRWAPGNPQIALEPWMYAKGDRPRVVITSGSRSVFIPALGLDFFRPLLGGSVLGGGDVEVIVAAPEPVAAQLRAEFPDVRAGFVPLDVVAPTADLVLHHGGGVTVMTLLNAGTPQLVLPEILASAIPMRPVDEFGASITLPSHTVPVEDTEAAVSKILGDTSYRAKAGELAAEIASMPSQAEVVKVIEGLV

>Kid24; N,N-dimethyltransferase

MYGAESAKFYHAQHTARGKDYRAEAETVVEQIQARLPGAASVLDVACGTGGHLPAFAELIGHTEGIDLSEPMLDIARTDLPHVPLHVGDMRSFDLGRTFDAVTCLFASIGYVSSRAELHETVRRLALHTNPSGVVVVEPWWFPETFIDRWVSSDVIEWEDTTVARATHTVREGNTSRMEVHYLQASPAGGVRYFTEVHRAMLFSREEYVEAFEEAGLRAEYVPEVQSRRGLFIGTRR
